# Anthocyanin Biosynthesis and Degradation Mechanisms in *Solanaceous* Vegetables: A Review

**DOI:** 10.3389/fchem.2018.00052

**Published:** 2018-03-09

**Authors:** Ying Liu, Yury Tikunov, Rob E. Schouten, Leo F. M. Marcelis, Richard G. F. Visser, Arnaud Bovy

**Affiliations:** ^1^Plant Breeding, Wageningen University and Research, Wageningen, Netherlands; ^2^Horticulture and Product Physiology, Wageningen University and Research, Wageningen, Netherlands; ^3^Graduate School Production Ecology & Resource Conservation, Wageningen University and Research, Wageningen, Netherlands

**Keywords:** anthocyanin biosynthesis, anthocyanin degradation, chemical structure, MYB transcription factor, discoloration, environmental regulation, light dependence, *Solanaceae*

## Abstract

Anthocyanins are a group of polyphenolic pigments that are ubiquitously found in the plant kingdom. In plants, anthocyanins play a role not only in reproduction, by attracting pollinators and seed dispersers, but also in protection against various abiotic and biotic stresses. There is accumulating evidence that anthocyanins have health-promoting properties, which makes anthocyanin metabolism an interesting target for breeders and researchers. In this review, the state of the art knowledge concerning anthocyanins in the *Solanaceous* vegetables, i.e., pepper, tomato, eggplant, and potato, is discussed, including biochemistry and biological function of anthocyanins, as well as their genetic and environmental regulation. Anthocyanin accumulation is determined by the balance between biosynthesis and degradation. Although the anthocyanin biosynthetic pathway has been well-studied in *Solanaceous* vegetables, more research is needed on the inhibition of biosynthesis and, in particular, the anthocyanin degradation mechanisms if we want to control anthocyanin content of *Solanaceous* vegetables. In addition, anthocyanin metabolism is distinctly affected by environmental conditions, but the molecular regulation of these effects is poorly understood. Existing knowledge is summarized and current gaps in our understanding are highlighted and discussed, to create opportunities for the development of anthocyanin-rich crops through breeding and environmental management.

## Introduction

Anthocyanins are an important class of flavonoids that represent a large group of plant secondary metabolites. Anthocyanins are glycosylated polyphenolic compounds with a range of colors varying from orange, red, and purple to blue in flowers, seeds, fruits and vegetative tissues (Tanaka and Ohmiya, 2008). As anthocyanins are water-soluble pigments that are mostly located in cell vacuoles, their hue, a color property, is influenced by the intravacuolar environment. Over 600 anthocyanins have been identified in nature (Smeriglio et al., 2016). In plants, the most common anthocyanins are the derivatives of six widespread anthocyanidins, namely pelargonidin, cyanidin, delphinidin, peonidin, petunidin, and malvidin (Kong et al., 2003). Anthocyanins protect plants against various biotic and abiotic stresses (Chalker-Scott, 1999; Ahmed et al., 2014), partially due to their powerful antioxidant properties. In addition, anthocyanin-rich food products have become increasingly popular due to their attractive colors and suggested benefits for human health (Pojer et al., 2013).

**Figure 1:**
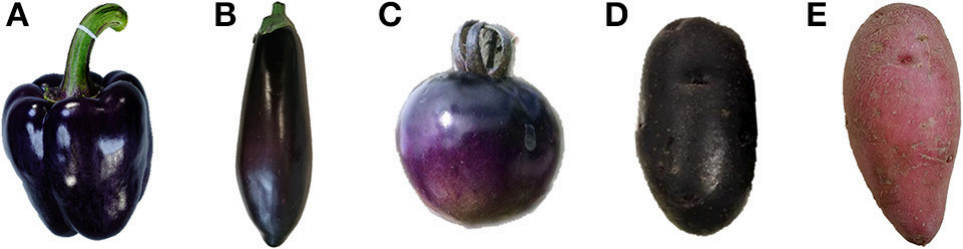
Example of *Solanaceous* vegetables rich in anthocyanins. **(A)** purple pepper fruit, **(B)** purple eggplant fruit, **(C)** purple tomato fruit, **(D)** purple potato tuber, **(E)** red potato tuber.

The *Solanaceae* contain many horticultural species of economic importance, including tomato (*Solanum lycopersicum*), pepper (*Capsicum* spp.), eggplant (*Solanum melongena*) and potato (*Solanum tuberosum*). Some of these *Solanaceae* produce anthocyanins (Dhar et al., 2015; Figure 1). In potato tubers, once produced, anthocyanins are stable; however, in purple-fruited genotypes of pepper and eggplant the abundance of anthocyanin levels are highest in unripe fruits and decrease upon ripening, often to complete disappearance. In this light, it is noteworthy that eggplant fruit reaches its commercial maturity long before its physiological ripeness (Mennella et al., 2012). Tomato fruits normally do not produce anthocyanins, but this trait can be obtained, either by genetic transformation or by introgression from several purple-fruited wild species. The latter can be achieved by combining the dominant *Anthocyanin fruit* (*Aft*) gene from *Solanum chilense* and the recessive *atroviolacea* (*atv*) gene from *S. cheesmaniae* into a cultivated tomato background (Povero et al., 2011; Maligeppagol et al., 2013). In general, anthocyanins accumulate in flowers, leaves, stems and fruits of *Solanaceae*, specifically in the peel of eggplant, pepper and tomato fruits as well as potato tubers, but also in the flesh of some potato genotypes (Matsubara et al., 2005; Lightbourn et al., 2008; Sapir et al., 2008).

Anthocyanin content depends on the balance between biosynthesis and degradation. Anthocyanin biosynthesis has been extensively studied, whereas knowledge regarding its degradation is limited (Holton and Cornish, 1995; Passeri et al., 2016). Genetic, developmental and environmental factors all regulate anthocyanin metabolism. This review discusses the state of the art concerning anthocyanin metabolism in four *Solanaceous* vegetables, i.e., tomato, pepper, eggplant, and potato. Firstly, the biochemistry and biological function of anthocyanins are elaborated and subsequently, the genetic and environmental regulation of both biosynthesis and degradation is discussed. In regard to overall research in the *Solanaceae*, the most extensive efforts to unravel anthocyanin metabolism have been undertaken in flowers of *Petunia hybrida* (Passeri et al., 2016), so when there is lack of information in these four vegetables, knowledge regarding petunia is used. The genetic mechanisms found in petunia appeared to be highly relevant for *Solanaceous* vegetables (Quattrocchio et al., 1999; Spelt et al., 2000). This review will be helpful in designing strategies for obtaining anthocyanin-rich crops via breeding and/or environmental control.

## Structural variation of anthocyanins in the main *Solanaceous* vegetables

**Figure 2:**
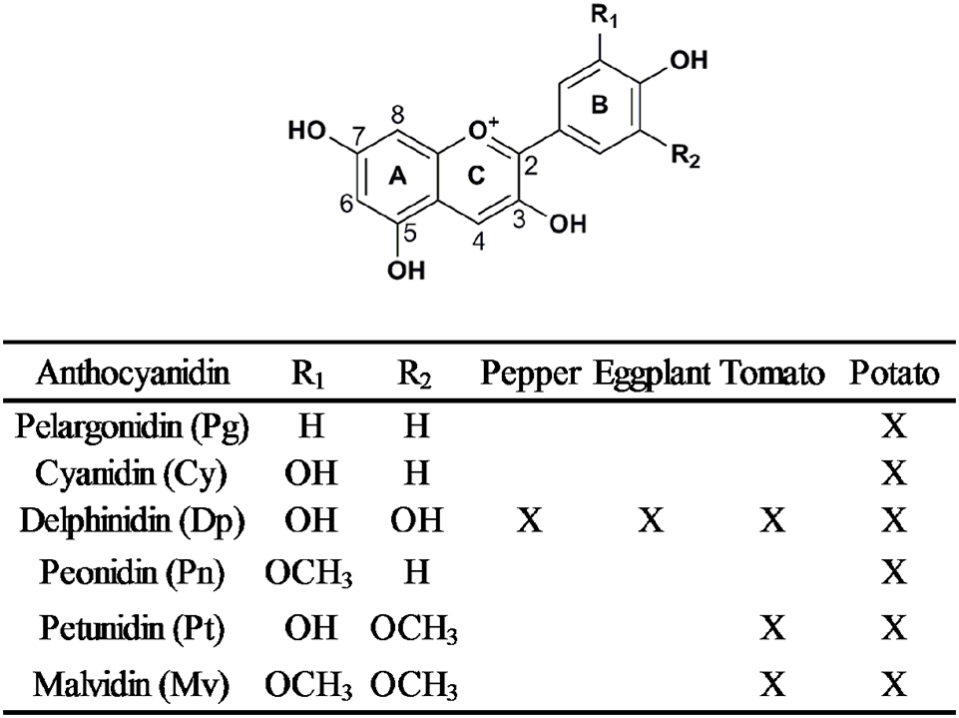
General chemical structure of anthocyanidins and the six most common anthocyanidins in *Solanaceous* vegetables, indicated by “X”.

Anthocyanins are a diverse class of flavonoids, which are composed of an anthocyanidin backbone with sugar and acyl conjugates (Stommel et al., 2009). Anthocyanidins are composed of two aromatic benzene rings separated by an oxygenated heterocycle (Tanaka et al., 2008; Figure 2). More than 20 anthocyanidins have been discovered, but only six of them are prevalent in plants (Zhao et al., 2014). Pelargonidin, cyanidin, and delphinidin are the primary anthocyanidins and differ from each other by the number of hydroxyl groups at their B-rings. They show orange/red, red/magenta and violet/blue hues, respectively (Tanaka and Ohmiya, 2008). Peonidin is derived from cyanidin by a single O-methylation, likewise, single or double methylation of delphinidin results in petunidin and malvidin, respectively (Figure 2). Besides the structure of anthocyanidin, the structure, quantity and position of conjugated sugar and acyl moieties also lead to anthocyanin diversification.

Delphinidin derivatives are the only anthocyanins identified in violet/black pepper and eggplant fruits. The most common anthocyanin structure in pepper and eggplant fruits is delphinidin-3-(*p*-coumaroyl-rutinoside)-5-glucoside (Azuma et al., 2008; Lightbourn et al., 2008; Stommel et al., 2009). The *p*-coumaroyl moiety could be substituted by either feruloyl or caffeoyl acyl moiety (Sadilova et al., 2006; Azuma et al., 2008). Acylated anthocyanins are the most abundant forms in pepper and eggplant, although in the latter some accessions are found in which a non-acylated anthocyanin, namely delphinidin-3-rutinoside, is predominant (Sadilova et al., 2006; Azuma et al., 2008; Toppino et al., 2016). Except for delphinidin-3-rutinoside, non-acylated anthocyanins account for only a small proportion of the total anthocyanin content. Despite the general structural similarity of anthocyanins in eggplant, deviations can be sometimes observed. For instance, in fruit peel of eggplant cv. Zi Chang, only two anthocyanins, delphinidin-3-glucoside-5-(coumaryl)-dirhamnoside and delphinidin-3-glucoside-5-dirhamnoside, are found in which position 3 carries a single glucose moiety instead of the common *p*-coumaroyl-rutinoside and position 5 is conjugated with a dirhamnosyl moiety (Zhang et al., 2014b). This suggests the existence of genetic variation for enzymes such as glycosyltransferases, which mediate the conjugation of anthocyanidins with sugar moieties.

As in pepper and eggplant, only delphinidin-based derivatives have been detected in purple tomato fruits. In fruits of an *Aft/Aft atv/atv* tomato genotype, delphinidin-3-rutinoside and petunidin-3-(*p*-coumaroyl-rutinoside)-5-glucoside are the major anthocyanins (Mes et al., 2008). In transgenic *SlANT1* tomato fruits that overexpress the main activator gene of the anthocyanin pathway, 3-rutinoside-5-glucoside conjugates of delphinidin, petunidin and malvidin, as well as their *p*-coumaroyl and caffeoyl acylated forms have been identified (Mathews et al., 2003). Additionally, in transgenic *Del/Ros1* tomato fruits, where two transcription factors that control the anthocyanin pathway in *Antirrhinum* are overexpressed, 3-(*p*-coumaroyl-rutinoside)-5-glucoside conjugates of delphinidin and petunidin are the main anthocyanins (Su et al., 2016). So, in contrast to pepper and eggplant, anthocyanins in purple tomato fruits can be methylated by the action of methyltransferases.

A larger structural variation of anthocyanins can be found in potato tubers. Throughout the large range of potato genotypes, the six most common anthocyanidins have all been identified. In red potato tubers, pelargonidin-3-(*p*-coumaroyl-rutinoside)-5-glucoside is the major anthocyanin with lower levels of peonidin-3-(*p*-coumaroyl-rutinoside)-5-glucoside and pelargonidin-3-(*trans*-feruloyl-rutinoside)-5-glucoside (Lewis et al., 1998; Naito et al., 1998). In purple potato tubers, petunidin-3-(*p*-coumaroyl-rutinoside)-5-glucoside is the predominant anthocyanin. In addition, 3-(*p*-coumaroyl-rutinoside)-5-glucosides of malvidin, peonidin, and delphinidin have been found in different purple cultivars. It is noteworthy that color deepening is strongly associated with increased levels of malvidin glycosides (Lewis et al., 1998; Lachman et al., 2012; Jiang Z. et al., 2016). Lachman et al. (2009) found that potato tubers of cv. British Columbia Blue contained almost exclusively cyanidin derivatives.

**Figure 3:**
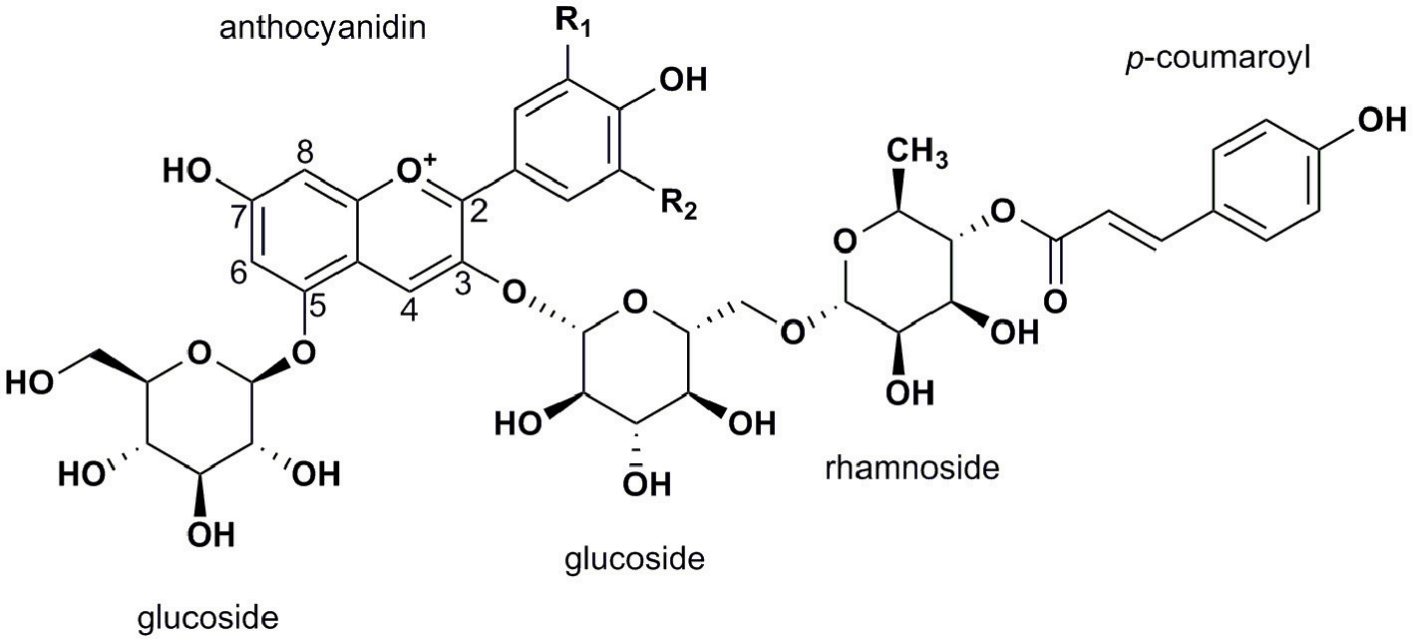
General structure of the most abundant anthocyanins in *Solanaceous* vegetables, anthocyanidin-3-(*p*-coumaroyl-rutinoside)-5-glucoside.

In summary, six common anthocyanidins have been discovered in *Solanaceous* vegetables. Delphinidin-based anthocyanins are the predominant structure in purple *Solanaceous* tissues and pelargonidin-based derivatives are the major structure in red potato tubers (Ichiyanagi et al., 2005; Sadilova et al., 2006; Mes et al., 2008; Lachman et al., 2012; Su et al., 2016). Despite the diverse anthocyanidin profiles observed in these four vegetables, the most common anthocyanin form is anthocyanidin-3-(*p*-coumaroyl-rutinoside)-5-glucoside (Figure 3).

## Biological function of anthocyanins

### Antioxidant activity

Anthocyanins have a higher antioxidant activity than other flavonoids, due to their positively charged oxygen atom (Kong et al., 2003; Figure 3). The antioxidant activity of anthocyanins depends on the degree of hydroxylation at the B-ring as well as the type and extent of acylation and glycosylation (Sadilova et al., 2006). Hydroxylation at the B-ring enhances antioxidant capacity (−OH > −OCH3 >> −H), therefore the antioxidant capacity of anthocyanidins decreases in the order of Dp > Pt > Mv = Cy > Pn > Pg (Pojer et al., 2013). Furthermore, glycosylation reduces the free radical scavenging ability of anthocyanins compared to their aglycone forms, by decreasing their hydrogen-donating, metal-chelating and electron delocalizing abilities (Zhao et al., 2014). The more sugar units at C3 and C5 position, the lower the antioxidant activity is (Sadilova et al., 2006). Finally, acylation of glycosyl moieties may partly circumvent the negative effect of glycosylation (Lachman and Hamouz, 2005). In summary, antioxidant activity increases with the number of hydroxyl groups in the B-ring and decreases with the number of glycosyl groups attached to the A and C ring. The latter effect is less severe when the glycosides are acylated.

### Benefits for plants

Anthocyanins play an important role in facilitating plant reproduction as they attract pollinators and seed dispersers by imparting bright colors (Harborne and Williams, 2000; Hoballah et al., 2007). In addition to their colorful characteristics, anthocyanins protect plants from several biotic and abiotic stresses (Chalker-Scott, 1999; Ahmed et al., 2014), which may provide them a better adaptation to climate change. Anthocyanins are photoprotective agents which shade and protect the photosynthetic apparatus by absorbing excess visible and UV light and scavenging free radicals (Guo et al., 2008). For instance, red pear fruits (cv. Anjou) and purple pepper leaves (cv. Huai Zi) rich in anthocyanins showed a more stable PS II photosynthetic capacity and a higher photo-oxidation tolerance compared to non-anthocyanin tissues (Li et al., 2008; Ou et al., 2013). Besides, anthocyanins often accumulate in young vegetative tissues and sun-exposed side of fruits to protect them from photoinhibition and photobleaching under light stress without significantly compromising photosynthesis (Steyn et al., 2002; Gould, 2003; Li and Cheng, 2008; Zhu et al., 2017). Moreover, the existence of colored anthocyanins can reduce the infestation of insects and pathogens. For example, anthocyanin-rich tobacco leaves were not preferred by the *Helicoverpa armigera* larvae. The mortality of *H. armigera* larvae was significantly increased and pupation of *Spodoptera litura* was significantly delayed by feeding anthocyanin-pigmented leaves, compared to controls fed with green ones (Malone et al., 2009). Anthocyanin-enriched tomato fruits exhibited lower susceptibility to gray mold (Zhang et al., 2013). Furthermore, transgenic tomato plants with higher anthocyanin content displayed an enhanced tolerance to heat stress (Meng et al., 2015). Wounded anthocyanin-rich leaf tissue showed faster recovery from oxidative stress caused by mecha nical injury (Gould et al., 2002).

Besides the protective effects during plant growth, anthocyanins may also play an important role to improve the postharvest performance of vegetables. For example, acting as antioxidants, anthocyanins prevent lipid peroxidation, and maintain membrane integrity to decelerate cell senescence (Jiao et al., 2012). Tomato fruits, enriched in anthocyanins, showed less over-ripening and a longer shelf-life (Bassolino et al., 2013; Zhang et al., 2013). The latter proposed a model explaining the extended shelf life of anthocyanin-rich tomatoes, as follows. Firstly, anthocyanins increase the antioxidant capacity of the fruit, which leads to suppression of both the activity and the signaling function of reactive oxygen species (ROS) and consequently may delay the processes of over-ripening. Secondly, anthocyanins increase fruit resistance to botrytis by altering the dynamics of the ROS burst generated by *Botrytis cinerea* infection, thereby limiting the induction of cell death required for growth and spreading of the fungus.

### Potential benefits for human health

Numerous *in vitro* and *in vivo* studies, including animal models, suggest that anthocyanins have health-promoting properties and may play a role in reducing chronic and degenerative diseases (Joseph et al., 2003; Lee et al., 2005; Achterfeldt et al., 2015; Charepalli et al., 2015).

Delphinidin derivatives, the main type of anthocyanins found in *Solanaceous* vegetables, have been associated with reduction of vascular inflammation and prevention of thrombosis (Watson and Schönlau, 2015). They may also protect the human skin from UV-B irradiance by inhibiting keratinocyte apoptosis. Potato anthocyanins repressed the reproduction of cell lines for human erythrocyte leukemia, stomach cancer and prostate cancer (Zhao et al., 2009). In addition, potato anthocyanins decreased the incidence of breast cancer in rats. Consumption of transgenic anthocyanin-rich tomatoes led to a 25% extension of the lifespan of the *p53* mouse, a cancer mouse model (Butelli et al., 2008), and a reduction in the development of atherosclerosis in a cardiovascular disease mouse model (Gonzali et al., 2009; Achterfeldt et al., 2015). The proliferation of human colon and ovarian cancer cell lines was significantly inhibited by peel extracts from purple tomatoes in a dose-dependent manner (Mazzucato et al., 2013). Although it is suggested that the antioxidant properties of anthocyanins form the basis for health benefits (Noda et al., 2000; Roleira et al., 2015), there is no evidence for health effects of antioxidants (Bast and Haenen, 2013; Carocho and Ferreira, 2013; Watson, 2013).

In contrast to the above-mentioned positive effects of anthocyanins, several studies reported no effect or even a negative effect of anthocyanins on health-related parameters (Tsuda, 2012; Pojer et al., 2013; Smeriglio et al., 2016). This apparent discrepancy may be due to differences in the anthocyanin composition and doses used and/or differences in experimental setup and methodology. Therefore, there is no unequivocal proof for the health benefits of anthocyanins.

## Anthocyanin biosynthetic mechanism

**Figure 4:**
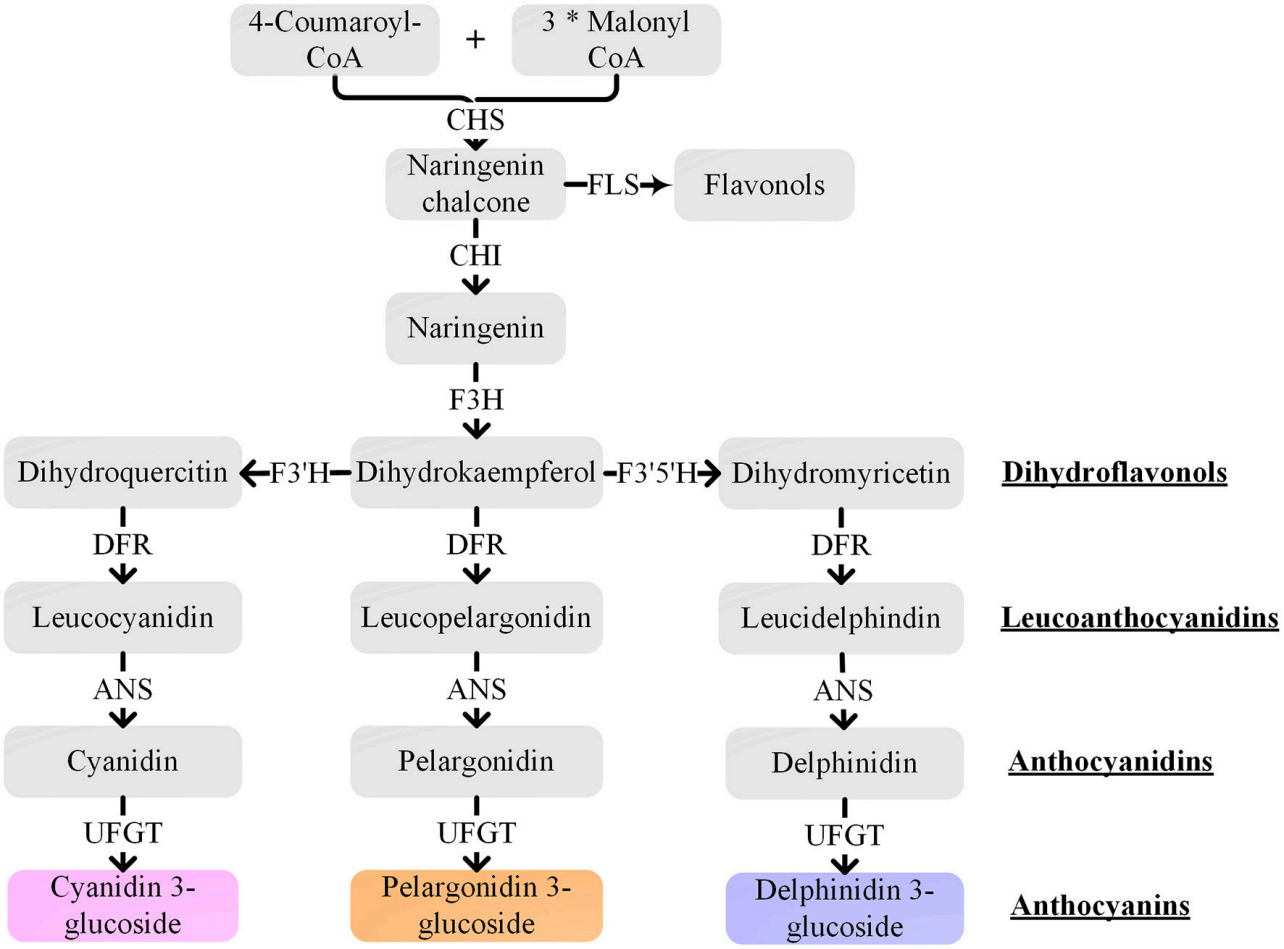
Schematic representation of the anthocyanin biosynthetic pathway. CHS, chalcone synthase; CHI, chalcone isomerase; F3H, flavanone 3-hydroxylase; F3′H, flavonoid 3′-hydroxylase; F3′5′H, flavonoid 3′,5′-hydroxylase; DFR, dihydroflavonol 4-reductase; ANS, anthocyanidin synthase; UFGT, flavonoid 3-O-glucosyltransferase; FLS, flavonol synthase. The “^*^” means multiplication.

The well-characterized anthocyanin biosynthetic pathway is a very conserved network in many plant species (Holton and Cornish, 1995; Tanaka and Ohmiya, 2008). The anthocyanin biosynthetic pathway (Figure 4) is an extension of the general flavonoid pathway, which starts with the chalcone synthase (CHS) mediated synthesis of naringenin chalcone from 4-coumaroyl-CoA and malonyl-CoA. Then, naringenin chalcone is isomerized by chalcone isomerase (CHI) to naringenin. Flavanone 3-hydroxylase (F3H) converts naringenin into dihydrokaempferol that can be further hydroxylated by flavonoid 3′-hydroxylase (F3′H) or flavonoid 3′,5′-hydroxylase (F3′5′H) into two other dihydroflavonols, dihydroquercetin or dihydromyricetin, respectively. Next, the three dihydroflavonols are converted into colorless leucoanthocyanidins by dihydroflavonol 4-reductase (DFR) and subsequently to colored anthocyanidins by anthocyanidin synthase (ANS). Finally, sugar molecules are attached to anthocyanidins by various members of the glycosyltransferase enzyme family, for instance, flavonoid 3-O-glucosyltransferase (UFGT), and might be further acylated with aromatic acyl groups by acyltransferases. CHS is the initial key enzyme of flavonoid biosynthesis. F3′H and F3′5′H are the primary enzymes responsible for the diversification of anthocyanins by determining their B-ring hydroxylation pattern and consequently their color (Tanaka and Brugliera, 2013). The substrate specificity of DFR also influences anthocyanin composition and pigmentation.

## Genetic regulation of anthocyanin biosynthesis

Expression of the regulatory and structural biosynthetic genes is the primary level at which the induction or shut down of anthocyanin biosynthesis in plants is regulated, although there are examples of post-transcriptional regulation of anthocyanin biosynthesis, for instance, the allele-specific substrate specificity of the DFR enzyme (Forkmann and Ruhnau, 1987). Structural genes encode the enzymes catalyzing each reaction step and, in dicot plants, can be divided into early (EBG) and late (LBG) biosynthetic genes (Dubos et al., 2010). Regulatory genes encode transcription factors that modulate the expression of the structural genes (Gonzali et al., 2009). The structural genes of the anthocyanin biosynthetic pathway function under control of a regulatory complex, called the MYB-bHLH-WD40 (MBW) complex, consisting of MYB, basic helix-loop-helix (bHLH) and WD40 repeat families.

### Early biosynthetic genes (EBGs)

EBGs—*CHS, CHI*, and *F3H* are the common flavonoid pathway genes which are involved in the biosynthesis of all downstream flavonoids. In general, the reported expression profile of EBGs varies and there is no consistent correlation between their expression levels and anthocyanin content in *Solanaceous* vegetables. In the anthocyanin-pigmented tomato *Aft/Aft* mutant (accession number LA1996), the expression of *SlCHS, SlCHI*, and *SlF3H* genes did not differ from that in the non-pigmented control genotype (Povero et al., 2011). In a different study, however, with fruits of the same LA1996, *SlCHS* was found to be substantially upregulated compared to red-fruited varieties (Sapir et al., 2008). A similar contrast has also been found in pepper fruits. Stommel et al. (2009) reported the upregulation of *CaCHS* in anthocyanin-pigmented fruits (*Capsicum annuum*, breeding line 06C59), while, Borovsky et al. (2004) and Aza-Gonzalez et al. (2013) found that the expression levels of *CaCHS, CaCHI*, and *CaF3H* during ripening of anthocyanin-pigmented fruits (*C. annuum* inbred line 5226, cv. Arbol and cv. Uvilla) were comparable to those of non-pigmented fruits (*C. chinense* PI 159234 and *C. annuum* cv. Tampiqueño 74). In eggplant, the expression level of *SmCHS* was reported to be significantly upregulated in black (cv. Black Beauty) or violet (cv. Classic) fruits compared to the green (genotype E13GB42) or white (cv. Ghostbuster) mutants (Stommel and Dumm, 2015; Gisbert et al., 2016). In addition, the transcript levels of *SmCHS* and *SmCHI*, but not *SmF3H*, correlated well with the anthocyanin accumulation pattern in cv. Lanshan Hexian (Jiang et al., 2016a). In potato tubers, the association of expression of EBGs and anthocyanin accumulation is more consistent. All EBGs were highly expressed in red (cv. AmaRosa and cv. Sullu) and purple tubers (cv. Guincho Negra, cv. W5281.2 and cv. Hei Meiren) and correlated with anthocyanin content (André et al., 2009; Jung et al., 2009; Payyavula et al., 2013; Liu Y. et al., 2015). Even in the same tuber, *StF3H* was found to be upregulated in anthocyanin-pigmented flesh compared to non-pigmented flesh (Stushnoff et al., 2010).

### Late biosynthetic genes (LBGs)

LBGs—*F3*′*H, F3*′*5*′*H, DFR, ANS*, and *UFGT* are required for the biosynthesis of specific classes of flavonoids, including anthocyanins. Positive correlations between expression levels of LBGs and anthocyanin content have been consistently observed in many *Solanaceous* vegetables (Borovsky et al., 2004; André et al., 2009; Povero et al., 2011; Aza-Gonzalez et al., 2013). In tomato fruits, *SlF3*′*5*′*H, SlDFR*, and *SlANS* showed a high expression in anthocyanin-pigmented mutants (*Aft/Aft, atv/atv* and *Aft/Aft atv/atv*) compared to their red-fruited controls (Sapir et al., 2008; Povero et al., 2011). During fruit development of anthocyanin-pigmented peppers (*C. annuum*), *CaF3*′*5*′*H, CaDFR, CaANS*, and *CaUFGT* were upregulated at a young fruit stage, reaching a maximum at the late unripe stage prior to ripening, and were downregulated afterwards, which corresponded to the transient anthocyanin accumulation pattern of these fruits (Borovsky et al., 2004; Stommel et al., 2009; Aza-Gonzalez et al., 2013). For eggplant, the expression levels of *SmDFR* and *SmANS* were significantly higher in black (cv. Black Beauty) or violet (cv. Classic) fruits compared to green (genotype E13GB42) or white (cv. Ghostbuster) fruited genotypes, respectively, at all fruit developmental stages, up to commercial ripeness (Stommel and Dumm, 2015; Gisbert et al., 2016). In anthocyanin-pigmented potato tuber skin, the *StF3*′*H, StF3*′*5*′*H* (Jung et al., 2005), *StDFR* (De Jong et al., 2003; Zhang et al., 2009), *StANS* and *StUFGT* were highly expressed (André et al., 2009; Jung et al., 2009; Liu Y. et al., 2015). *StDFR* was also upregulated in red and purple tuber flesh (Stushnoff et al., 2010).

Transcription of structural genes involved in the anthocyanin biosynthetic pathway has many similarities in *Solanaceous* vegetables. In summary, the EBGs are expressed to a sufficient level in both anthocyanin-pigmented and non-pigmented tissues. Generally, there is no consistent correlation between their expression levels and anthocyanin content, which is most likely due to the fact that expression of EBG's is not only required for the production of anthocyanins, but also for that of other flavonoids, such as flavonols or flavanones. In contrast, the transcript level of LBGs coincides well with anthocyanin content and is significantly higher in pigmented compared to non-pigmented tissues, suggesting that variations in LBG expression determine the quantitative variation of anthocyanins in *Solanaceous* vegetables (Table 1). In fruits of pepper, eggplant and tomato, the expression of LBGs have a very similar, ripening dependent pattern, suggesting the presence of a conserved regulatory machinery that coordinates their expression.

**Table 1:** Overview of the correlations between the expression of anthocyanin structural genes and anthocyanin content in tomato, pepper, and eggplant fruits and potato tubers.

**Genes**	**Tomato**	**Pepper**	**Eggplant**	**Potato**
*CHS*	±	±	+	+
*CHI*	–	–	+	+
*F3H*	–	–	–	+
*F3′H*				+
*F3′5′H*	+	+		+
*DFR*	+	+	+	+
*ANS*	+	+	+	+
*UFGT*		+		+

*“+” means there is a quantitative association in published data, “−” means no association, “±” means studies provide conflicting data. Information about tomato is based on Sapir et al. (2008) and Povero et al. (2011). Information about pepper is based on Borovsky et al. (2004), Stommel et al. (2009), and Aza-Gonzalez et al. (2013). Information about eggplant is based on Stommel and Dumm (2015), Gisbert et al. (2016), and Jiang et al. (2016a). Information about potato is based on De Jong et al. (2003), Jung et al. (2005), André et al. (2009), Jung et al. (2009), Zhang et al. (2009), Stushnoff et al. (2010), Payyavula et al. (2013), and Liu Y. et al. (2015)*.

### Regulatory genes

The anthocyanin biosynthetic pathway is transcriptionally regulated by a MBW complex. The MYB transcription factors primarily determine the activation or repression role of the MBW complex, by binding to the promoters of structural genes, together with the common bHLH and WD40 factors. The MYB activators are mainly from the R2R3-MYB family. Known repressors consist of both R2R3-MYB and R3-MYB transcription factors. The expression of the *R2R3-MYB* and *bHLH* regulatory genes, is specific for pigmented tissue in most cases (Koes et al., 2005), while that of *WD40s*, which are involved in stabilizing the MBW complexes, is generally similar between anthocyanin-pigmented and non-pigmented tissues (Koes et al., 2005; Ramsay and Glover, 2005). Regulatory genes encoding MYB, bHLH, and WD40 transcription factors in tomato, pepper, eggplant, potato, and petunia are summarized in Table 2. Genes encoding MYB repressors have not been identified in tomato, pepper, eggplant, and potato yet.

**Table 2:** Regulatory genes encoding R2R3-MYB, bHLH, and WD40 transcription factors in tomato, pepper, eggplant and potato and their corresponding orthologs in petunia.

**Regulatory Genes**	**Tomato**	**Pepper**	**Eggplant**	**Potato**	**Petunia**
*R2R3-MYB* activator	*SlANT1, SlAN2*	*CaMYB_*A*_*	*SmMYB1, SmMyb_*C*_*	*StAN1, StMtf, StMYBA1*	*PhAN2*
*R2R3-MYB* repressor	–	–	–	–	*PhMYB27*
*R3-MYB* repressor	–	–	–	–	*PhMYBx*
*bHLH (AN1)*	*SlAN1*	*CabHLH*	*SmbHLH*	*StbHLH1*	*PhAN1*
*bHLH (JAF13)*	*SlJAF13*	–	–	*StJAF13*	*PhJAF13*
*WD40*	*SlAN11*	*CaWD40*	*SmWD40*	*StAN11, StWD40*	*PhAN11*

It is important to note that the nomenclature of orthologous genes in the different species is not consistent. For example, the potato *AN1* gene encodes an R2R3 MYB transcription factor, while its orthologs in petunia and tomato are called *AN2*. Furthermore, in the latter two species *AN1* encodes a bHLH transcription factor. Since petunia is the best-studied model for anthocyanins, the petunia nomenclature is used when discussing general principles.

#### R2R3-MYB activators

The R2R3-MYB activator is a key element in the MBW complex that determines upregulation of anthocyanin biosynthesis. Genes encoding R2R3-MYB activators of *Solanaceous* vegetables are orthologs of the petunia *PhAN2* (Payyavula et al., 2013; Docimo et al., 2016). In transgenic tomato fruits, overexpression of two *MYB* genes, *SlANT1* or *SlAN2*, led to accumulation of anthocyanins and up-regulation of EBGs, LBGs and the *bHLH* gene *SlAN1*, but not the *bHLH* gene *SlJAF13*, nor the *WD40* gene *SlAN11* (Mathews et al., 2003; Kiferle et al., 2015; Meng et al., 2015). The *SlANT1* and *SlAN2* were both proposed to be candidates for the *Aft/Aft* mutation (Povero et al., 2011) and later Schreiber et al. (2012) reported that *SlANT1* rather than *SlAN2* was the gene responsible for anthocyanin production in the *Aft/Aft* genotype, since the *SlANT1* showed the best genetic linkage with the *Aft/Aft* mutation. The *SlANT1* revealed both nucleotide and amino acid polymorphisms between the *Aft/Aft* and cultivated genotypes. The overexpressed *SlANT1*^*c*^ originating from *S. chilense* was more efficient than the overexpressed *SlANT1*^*l*^ from *S. lycopersicum* in enhancing transcript levels of *SlF3H, SlDFR*, and *SlANS* as well as in increasing anthocyanin content, suggesting that not only the expression of *SlANT1*, but also structural differences in SlANT1 protein between these two species affected the induction of anthocyanin biosynthesis. In pepper, the dominant *CaMYB*_*A*_ gene was uniquely expressed in purple fruits (*C. annuum* breeding lines 5226 and 06C59) and closely associated with anthocyanin accumulation (Borovsky et al., 2004; Stommel et al., 2009). As the coding regions of *CaMYB*_*A*_ between purple- (5226) and green-fruited (*C. chinense* PI 159234) genotypes were identical, the lack of expression of *CaMYB*_*A*_ in green-fruited genotypes was probably due to variations in their promoter regions. On one hand, Borovsky et al. (2004) only found a correlation between expression of *CaMYB*_*A*_ and expression of LBGs (*CaDFR* and *CaANS*), rather than that of EBGs. On the other hand, transient VIGS silencing of *CaMYB*_*A*_ effectively down-regulated the expression of both EBGs and LBGs and led to reduced anthocyanin content (Aguilar-Barragán and Ochoa-Alejo, 2014). This suggests that, in addition to regulating the expression of LBGs (*CaF3*′*5*′*H, CaDFR*, and *CaUFGT*), CaMYB_A_ transcription factor can regulate the expression of some EBGs (*CaCHS* and *CaF3H*) as well. In addition, the expression of EBGs is also influenced by other transcription factors in the flavonoid pathway, e.g., SlMYB12 in tomato (Ballester et al., 2010), and this may explain the weak correlation between EBG expression and anthocyanin formation. In eggplant, *SmMYB1* and *SmMyb*_*C*_ displayed higher transcript levels in anthocyanin-pigmented fruits compared to non-pigmented fruits (Zhang et al., 2014b; Stommel and Dumm, 2015; Gisbert et al., 2016). The significant upregulation of *SmMYBs* was in accordance with the elevated expression level of structural genes and anthocyanin content. In potato, *StAN1*, previously named *StAN2* in some studies, was highly expressed in anthocyanin-pigmented tubers and displayed a positive correlation with the transcript levels of structural genes, as well as with anthocyanin content (André et al., 2009; Jung et al., 2009; Payyavula et al., 2013). In addition, overexpression of *StAN1* under the control of *CaMV* 35S promoter in transgenic potato plants resulted in anthocyanin accumulation in tuber skin and flesh, suggesting its key role in regulating anthocyanin biosynthesis in tubers (Jung et al., 2009). Moreover, among different colored tubers, there are variations in the number of repeats of a 10-amino acid motif in the C-terminus of StAN1. Through functional analysis in tobacco leaves, the presence of only one copy of this 10-amino acid motif appeared optimal for activating anthocyanin production (Liu et al., 2016). To sum up, the R2R3-MYB activator, as part of the MBW complex, is able to upregulate the expression of both EBGs and LBGs in *Solanaceae*. In addition, their expression is always positively correlated with that of LBGs. Furthermore, the functional efficiency of R2R3-MYB transcription factors is determined by variations in their amino acid sequences.

#### bHLH transcription factors

In the MBW complex, the bHLH transcription factors determine the specificity in recognizing transcription factor binding sites in the target gene promoters and activating transcription (Montefiori et al., 2015). In *Solanaceae*, there are two main bHLH clades involved in the regulation of anthocyanin biosynthesis, which are orthologs of petunia PhAN1 and PhJAF13. It was suggested that they could not be mutually exchanged and participated in different steps of the anthocyanin regulatory cascade (Spelt et al., 2000). In pepper and eggplant, substantially higher transcript levels of *CabHLH* and *SmbHLH*, orthologs of *PhAN1*, have been found in anthocyanin-pigmented fruits compared to non-pigmented ones (Stommel et al., 2009; Stommel and Dumm, 2015; Gisbert et al., 2016). This upregulation of *CabHLH* and *SmbHLH* correlated positively with elevated expression levels of structural genes and anthocyanin content. Overexpression of tomato *SlAN1* greatly elevated anthocyanin content in tomato fruit peel (Qiu et al., 2016). *SlAN1* has been suggested to directly regulate (as part of the MBW complex) the expression of *SlF3*′*5*′*H* and *SlDFR* as they were always co-expressed (Spelt et al., 2000; Qiu et al., 2016). The potato *StbHLH1*, an ortholog of *PhAN1*, was highly expressed in red and purple tubers (Payyavula et al., 2013). A transcriptomics study with white and purple potato tubers revealed that expression of *StbHLH1* alone was not sufficient to regulate anthocyanin biosynthesis and obtain purple pigmentation (Liu Y. et al., 2015). *StbHLH1* was involved in anthocyanin regulation in both tuber peel and flesh, with activation by StJAF13 (Liu Y. et al., 2015; Liu et al., 2016). The MYB transcription factor can form a complex with two bHLH clades, separately. For example, interactions between StAN1 (R2R3-MYB ortholog of PhAN2) and StbHLH1 or StJAF13 have been confirmed using yeast two-hybrid assays (D'Amelia et al., 2014). D'Amelia et al. (2014) found that transformation of either *StAN1* together with *StbHLH1* or *StJAF13* in tobacco resulted in a more intense purple pigmentation than in case of *StAN1* alone.

**Figure 5:**
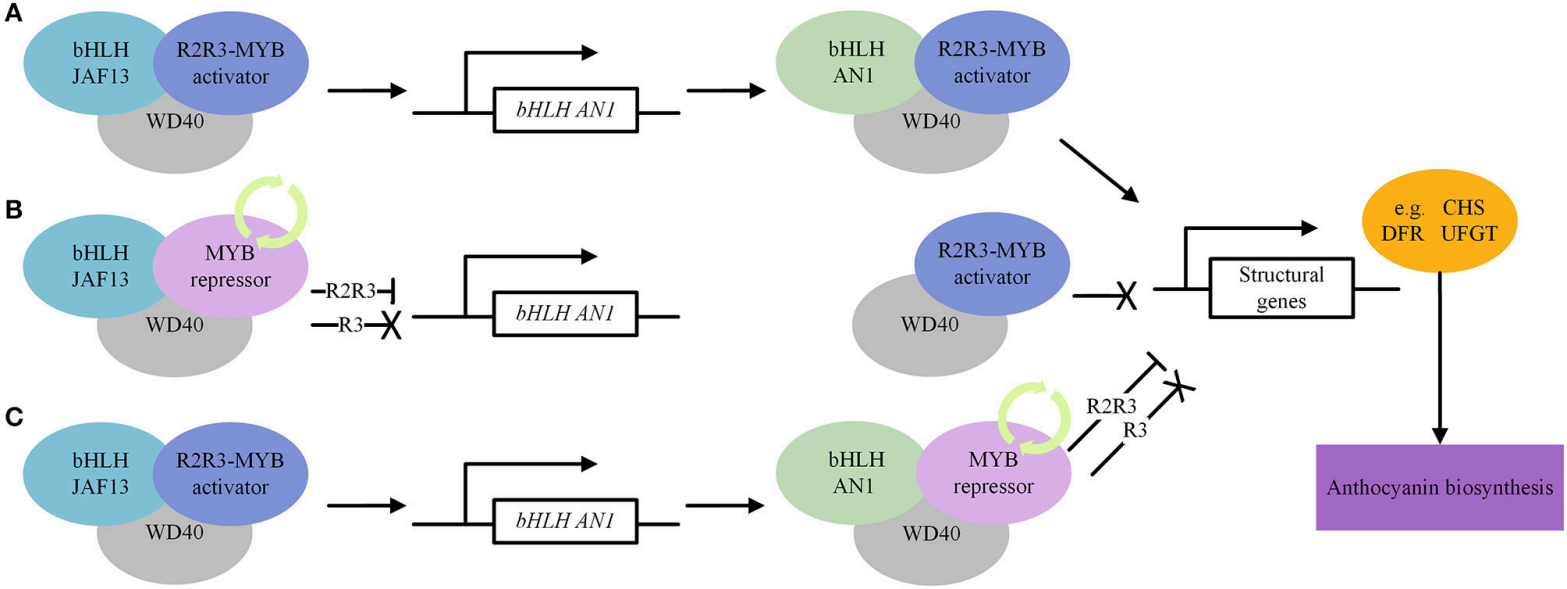
A simplified model depicting the regulatory mechanism of transcription factors, MYB, bHLH and WD40, that modulate the expression of structural genes of the anthocyanin biosynthetic pathway. **(A)** Activate regulation of anthocyanin biosynthesis. **(B)** Repressive regulation of anthocyanin biosynthesis. MYB repressors compete with MYB activators for bHLH JAF13. **(C)** Repressive regulation of anthocyanin biosynthesis. MYB repressors compete with MYB activators for bHLH AN1. The “ → ” means activation, “—|” means repression and “X” means inactivation.

In general, AN1 directly activates the anthocyanin biosynthetic pathway through the MYB-AN1-WD40 complex, whereas JAF13 regulates the pathway indirectly, by regulating *AN1* transcription through the MYB-JAF13-WD40 complex upstream in the regulatory cascade (Montefiori et al., 2015; Figure 5A).

#### WD40 transcription factors

WD40 proteins provide a stable platform for MYB and bHLH proteins to form the MBW complex together. Generally, the expression level of *WD40* genes, for instance, eggplant *SmWD40*, pepper *CaWD40* and potato *StAN11*, was comparable between anthocyanin-pigmented and non-pigmented tissues (Stommel et al., 2009; Liu Y. et al., 2015; Stommel and Dumm, 2015). Their expression levels hardly changed with altered transcript levels of structural genes or anthocyanin content (Stommel and Dumm, 2015). In addition to naturally pigmented plants, the expression level of tomato *SlAN11* in peel was similar between wild type and anthocyanin-rich 35S:*SlANT1* transgenic plants (Kiferle et al., 2015). This indicates that a basal expression level of *WD40* might be sufficient to facilitate anthocyanin production in *Solanaceous* vegetables. Despite its constant expression, WD40 is an indispensable transcription factor for anthocyanin biosynthesis. For example, a mutation in *PhAN11* in petunia line W137 resulted in white flowers (Quattrocchio et al., 2006). Additionally, in pepper fruits whose *CaMYB*_*A*_ and *CaWD40* genes were silenced independently by VIGS, a similar reduction in transcript levels of structural genes as well as anthocyanin content was revealed (Aguilar-Barragán and Ochoa-Alejo, 2014). There are few exceptions for the upregulation of *WD40* genes. The expression of *SmWD40* in eggplant cv. Black Beauty increased several-fold exclusively when fruits reached the market stage (Gisbert et al., 2016). A potato *StWD40* was significantly up-regulated in red and purple fleshed tubers, together with *StAN1* and *StbHLH1*. The *StWD40* is the first potato *WD40* gene whose expression is associated with anthocyanin content (Payyavula et al., 2013). However, the reason for their upregulation is still unclear.

#### MYB repressors

In *Solanaceae*, MYB repressors are barely known, only a few studies revealed pieces of information in petunia (Table 2). Two categories of MYB transcription factors, R2R3-MYB and R3-MYB repressors, have been shown to downregulate anthocyanin biosynthesis (Albert et al., 2014). The R2R3-MYB repressors contain a repression motif in their C terminus, while R3-MYB repressors do not. In general, both types of MYB repressors are able to passively repress anthocyanin biosynthesis by competing with MYB activators for coupling to bHLH proteins in the MBW complex thereby reducing its activation capability. In addition, the R2R3-MYB repressors turn the function of the MBW complex from activation to repression through their repression motif which leads to active suppression of the transcription of downstream genes. For example, the petunia PhMYB27, an R2R3-MYB repressor, incorporated or bound to the MBW complex and suppressed anthocyanin biosynthesis through its C-terminal EAR motif by binding to the promoter of target genes. This not only impaired the expression of structural genes but also that of *PhAN1*. R3-MYB repressors cannot directly target genes due to the lack of a repression motif, thus they can only exhibit passive suppression by reducing the pool of MBW activation complexes that can bind to the promoters of biosynthetic genes. For example, overexpression of *AtCPC*, an R3-MYB repressor (Matsui et al., 2008; Zhu et al., 2009; Albert et al., 2014), in transgenic tomato plants caused downregulation of anthocyanin structural genes and inhibited anthocyanin biosynthesis (Wada et al., 2014). PhMYBx, a petunia homolog of AtCPC, inhibited anthocyanin synthesis by binding to PhAN1 and PhJAF13 (Koes et al., 2005).

#### A regulatory mechanism model

By linking the related information together, we hypothesized a model in Figure 5 that describes the regulatory mechanism of anthocyanin biosynthesis. The R2R3-MYB activator first interacts with JAF13 (bHLH) and WD40 and forms a MYB-JAF13-WD40 complex to activate transcription of *AN1 (bHLH)*. Subsequently, the R2R3-MYB activator binds to AN1 and WD40 to form a MYB-AN1-WD40 activation complex to positively regulate anthocyanin biosynthesis (Figure 5A). The MYB repressors compete with MYB activators for binding to JAF13 and AN1, thereby reducing the number of MBW activation complexes. As a consequence, JAF13, in the inactive R3-MYB-JAF13-WD40 complex, loses its ability to upregulate the expression of *AN1*, leading to a reduction of the AN1 component in the MBW activation complex. In addition, the R2R3-MYB-JAF13-WD40 repressive complex suppresses transcription of the *AN1* gene through the suppression motif of R2R3-MYB repressors, which leads to a further reduction of the AN1 component. The inactive R3-MYB-AN1-WD40 complex loses its capacity to regulate anthocyanin biosynthesis, while the R2R3-MYB-AN1-WD40 repressive complex actively inhibits the transcription of target structural genes (Figures 5B,C).

Besides the MYB repressors, microRNAs (miRNA) have also been found to downregulate anthocyanin biosynthesis at the post-transcriptional level. For instance, miRNA858 suppressed the expression of R2R3-MYB activators in tomato (Jia et al., 2015).

## Anthocyanin discoloration mechanisms

Discoloration and color-changing phenomena have been observed in plant tissues during development (Oren-Shamir, 2009). Anthocyanin discoloration might be due to either anthocyanin reduction in plant tissues or to structural changes of the anthocyanin molecule that leads to a loss of color. The latter has only been shown *in vitro*, where a change in pH from acidic to neutral can lead to a complete, though reversible, discoloration of the anthocyanin molecule due to the formation of colorless isoforms (Basílio and Pina, 2016). Although not yet reported, we cannot exclude that this may also happen *in planta*. Discoloration due to a reduction in anthocyanin concentration is more common (Borovsky et al., 2004). This could simply result from a dilution effect caused by cell expansion during growth. However, such dilution effect is unlikely to play a significant role in anthocyanin discoloration in *Solanaceous* vegetables, since anthocyanin related pigmentation, e.g., in pepper and eggplant, begins to vanish at later stages of fruit development when fruits have almost reached their maximum size. Anthocyanin discoloration in *Solanaceae* is therefore more likely due to a change in the balance between anthocyanin biosynthesis and degradation, i.e., a decrease or termination of anthocyanin biosynthesis and/or an increase of anthocyanin degradation. There are various enzymatic and non-enzymatic factors that affect the stability and concentration of anthocyanins, which for the sake of simplicity, we call them degradation factors. In contrast to biosynthesis, anthocyanin degradation mechanisms have been much less studied and understood, though there is accumulating evidence supporting *in planta* degradation of anthocyanins (Oren-Shamir, 2009; Zipor et al., 2015; Movahed et al., 2016; Passeri et al., 2016; Niu et al., 2017). Below we discuss mechanisms that can lead to anthocyanin discoloration in *Solanaceous* crops. We also include mechanisms observed in other species, due to limited information in *Solanaceae*.

### Downregulation of anthocyanin biosynthesis

**Figure 6:**
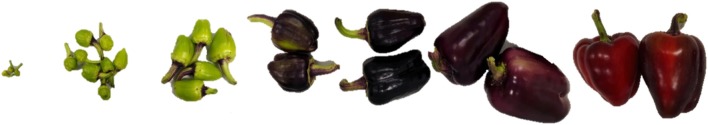
Anthocyanin accumulation and discoloration profile in pepper fruits of cv. Tequila during fruit development.

Anthocyanin levels are the net result of biosynthesis and degradation. A shift toward degradation, which is caused by downregulation of anthocyanin biosynthesis, leads to a decrease in anthocyanin content and, eventually, disappearance. In fruits of tomato, eggplant, and pepper, anthocyanins often accumulate in the skin of unripe fruits and afterwards their levels decrease during ripening (Borovsky et al., 2004; Povero et al., 2011; Mennella et al., 2012). In purple pepper fruits (Figure 6), anthocyanin discoloration was accompanied with a decline in expression of positive regulatory genes, such as *CaMYB*_*A*_, and most of its downstream structural genes, leading to a reduced anthocyanin biosynthesis relative to its degradation (Borovsky et al., 2004). When positive transcription factors of the anthocyanin pathway were constitutively overexpressed, as in transgenic *Del*/*Ros1* tomato, anthocyanins accumulated during all ripening stages resulting in a deep purple ripe fruit (Maligeppagol et al., 2013). Kiferle et al. (2015) separately overexpressed two similar tomato MYB genes, *SlANT1* and *SlAN2*, under control of the constitutive *CaMV* 35S promoter. In both cases, this led to intense anthocyanin pigmentation in immature fruits. However, this intense pigmentation was only maintained in 35S:*ANT1* mature fruits, whereas anthocyanins partially degraded in 35S:*SlAN2* mature fruits. Thus, a decrease in the expression of anthocyanin activators plays an important role in reducing anthocyanin biosynthesis. Even upon constitutive overexpression of anthocyanin activators, the final anthocyanin concentration in ripe fruits may depend on which regulatory gene is overexpressed and their abilities to activate downstream structural genes.

### Enzymatic factors influencing anthocyanin degradation

Anthocyanin discoloration may occur as a result of active enzyme-driven breakdown processes. The active enzymatic *in planta* degradation of anthocyanins was first suggested in *Brunfelsia calycina* (*Solanaceae*) flowers whose color changed rapidly from dark purple to complete white after opening (Vaknin et al., 2005). Later, a vacuolar class III peroxidase, BcPrx01, was suggested to be responsible for this anthocyanin degradation (Zipor et al., 2015).

**Figure 7:**
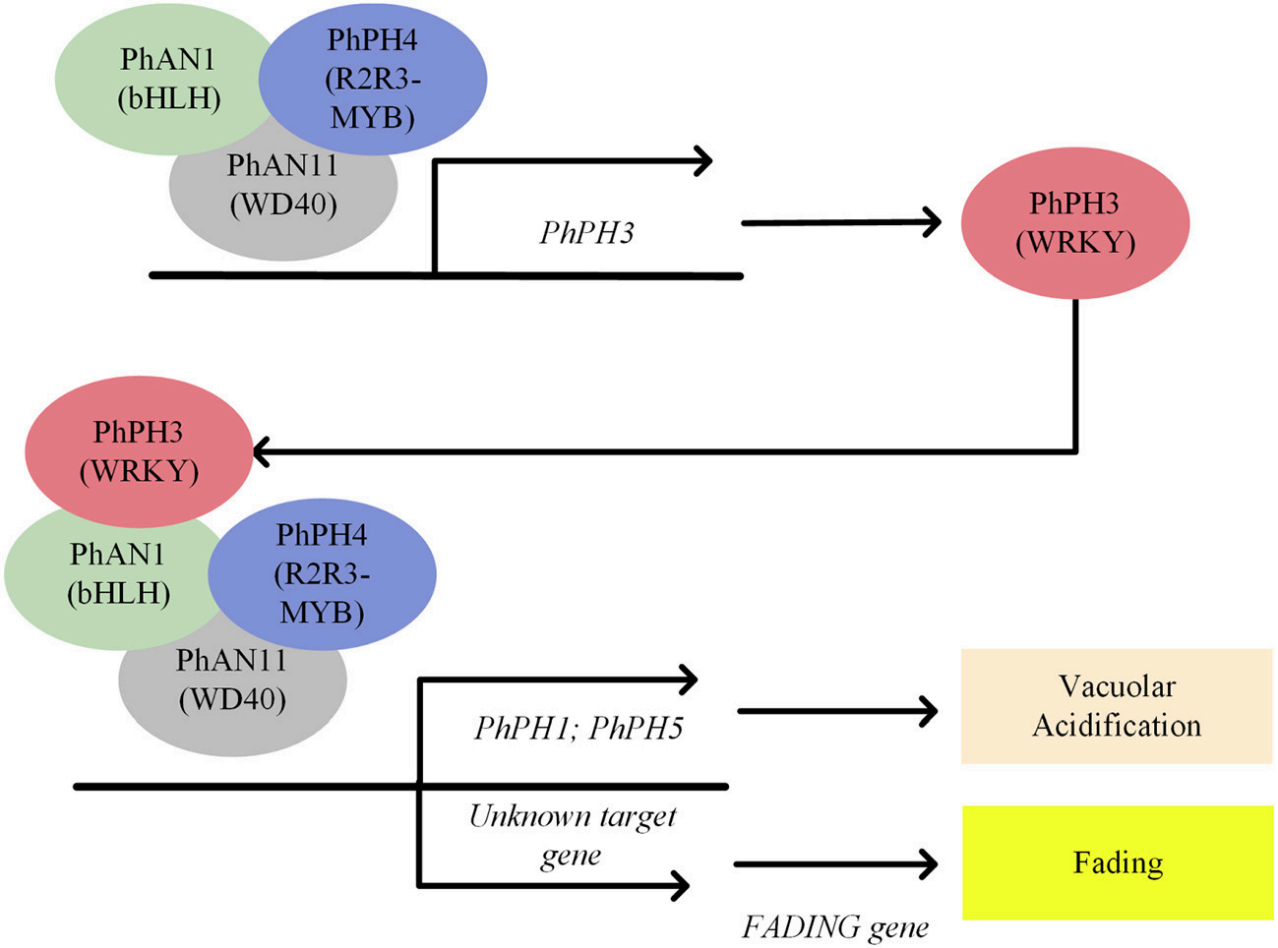
A schematic model of transcriptional regulation of vacuolar acidification and color fading in petunia petals under control of the PhPH4-PhAN1-PhAN11-PhPH3 complex.

Additional evidence for active enzymatic anthocyanin degradation was obtained from a petunia mutant whose petal color completely faded after bud opening (Quattrocchio et al., 2006). Color fading in petunia has a strong substrate specificity for anthocyanidin-3-(*p*-coumaroyl-rutinoside)-5-glucoside, which is the most common anthocyanin in *Solanaceous* vegetables (De Vlaming et al., 1982). This color fading only occurs in a genetic background containing a dominant *FADING* (*FA*) gene, which has not been cloned yet. However, the *FA* gene is not the only precondition for fading, since the fading effect of FA is restricted to certain petunia backgrounds with bluish petal colors (Quattrocchio et al., 2006). The bluish anthocyanin color is due to an increased vacuolar pH, suggesting that the FA action might be pH-dependent. In petunia, vacuolar pH is regulated by an MBW complex consisting of PhPH4 (R2R3-MYB)-PhAN1 (bHLH)-PhAN11(WD40) plus the WRKY transcription factor PhPH3. The PhPH4-PhAN1-PhAN11-PhPH3 complex regulates the expression of two proton pumps (PhPH1 and PhPH5) responsible for acidification of the vacuole, leading to a red hue of the anthocyanins. Mutant analysis revealed that the fading effect was not dependent on the vacuolar pH, since Phph1 and Phph5 mutants in an *FA* background had an increased vacuolar pH, but did not show any fading phenotype (Verweij et al., 2008, 2016; Faraco et al., 2014). In contrast, mutations in the PhPH4-PhAN1-PhAN11-PhPH3 complex regulating vacuolar pH (*Phph3, Phph4* and *Phan1)* revealed a clear fading phenotype (Quattrocchio et al., 2006; Passeri et al., 2016), suggesting that misregulation of unidentified downstream genes of the PhPH4-PhAN1-PhAN11-PhPH3 complex is an essential component for color fading. The unknown target genes might counteract the FA action to protect anthocyanins from degradation, or, alternatively, might actively repress expression of the *FA* gene to ensure anthocyanin stability (Figure 7).

In blood orange and litchi fruits, β-glucosidase and polyphenol oxidase and/or peroxidase have been suggested to be involved in anthocyanin degradation during the final ripening stage (Zhang et al., 2001, 2005; Barbagallo et al., 2007). Oren-Shamir (2009) proposed three candidate enzyme families: polyphenol oxidase, peroxidase and β-glucosidases, to be involved in anthocyanin degradation. There are two presumed anthocyanin degradation pathways. One is the direct oxidation by peroxidase. The other is comprised by a two-step degradation, deglycosylation by β-glucosidase and oxidation by polyphenol oxidase or peroxidase (Barbagallo et al., 2007; Oren-Shamir, 2009).

### Non-enzymatic factors influencing anthocyanin color and stability

Besides enzymatic factors, non-enzymatic factors also affect anthocyanin color and stability, and may enhance their vulnerability to enzymes that degrade anthocyanins. The chemical structure of anthocyanin determines its color and stability. The higher the level of B-ring hydroxylation, the more purple the color, but the more unstable the anthocyanins are (Woodward et al., 2009). The effect of glycosylation varies depending on the number and the position of sugar moieties (Zhang et al., 2014a). Glycosylation at C3 elevates stability and shifts color slightly toward red. The stabilizing effect of diglycosides at C3 is stronger than that of monoglycosides. In contrast, glycosylation at C5 reduces pigment intensity. Acylation increases anthocyanin stability and an increasing number of acyl moieties causes a color shift from red to blue (Lachman and Hamouz, 2005). Co-pigmentation, normally with flavones, flavonols or anthocyanins, results in more stable and intensely colored anthocyanins that shift color toward blue (Zhang et al., 2014a). Metal ions, for example, iron and magnesium, improve anthocyanin stability by forming complexes with them (Oren-Shamir, 2009). Furthermore, anthocyanins show pH-dependent structural isoforms in acidic and neutral solutions, but degrade in alkaline environments (Woodward et al., 2009). In the acidic vacuole, the color of anthocyanins shifts from red to blue with increasing pH. For example, the color of petunia mutants with an increased vacuolar pH (from around 5.5 to 6.0) shifted from red to blue (Quattrocchio et al., 2006).

## Environmental regulation of the anthocyanin pathway

Anthocyanin metabolism can be influenced by environmental factors. For instance, high irradiance (Lightbourn et al., 2007), UV/blue light (Guo and Wang, 2010; Jiang et al., 2016b), and low temperature (Qiu et al., 2016) promoted anthocyanin biosynthesis while high temperature induced its degradation (Movahed et al., 2016).

### Light

Light is one of the most important environmental factors affecting anthocyanin accumulation. High light intensity stimulates anthocyanin production in many plant species (Maier and Hoecker, 2015). For example, the part of the tomato fruit (*Aft/Aft atv/atv*) surface directly exposed to light showed a more intense anthocyanin pigmentation compared to the shaded parts (Mazzucato et al., 2013). In addition to intensity, light quality also affects anthocyanin biosynthesis. Poor anthocyanin pigmentation of eggplant fruits, growing in a greenhouse with low UV transmittance, was improved by providing UV-A irradiation (Matsumaru et al., 1971). Guo and Wang (2010) reported UV-A irradiation increased anthocyanin content in tomato seedlings compared to white light. They also suggested that UV-A radiation on tomato fruits increased their anthocyanin content. Blue and red light have also been reported to induce anthocyanin biosynthesis compared to darkness (Xu et al., 2014; Liu Z. et al., 2015). The amount of anthocyanin in tomato seedlings was elevated with an increased percentage of blue light (Hernández et al., 2016). For supplemental far-red light, contradictory effects on anthocyanin content have been reported (Li and Kubota, 2009; Liu Z. et al., 2015).

The effects of light intensity and spectrum on anthocyanin content are attributed to their influence on anthocyanin biosynthetic genes. Albert et al. (2009) suggested that high-light regulated anthocyanin production mainly through controlling R2R3-MYB transcription factors. *Solanaceous* R2R3-MYB activators such as SlAN2 and CaMYB_A_, were upregulated by high light, whereas an R2R3-MYB repressor, PhMYB27, was downregulated (Lightbourn et al., 2007; Albert et al., 2011; Kiferle et al., 2015). Transcription levels of *Solanaceous JAF13* and *AN11* were not affected by high irradiance (Lightbourn et al., 2007; Albert et al., 2014; Kiferle et al., 2015). The reported effect of high light on transcription of *Solanaceous AN1* was not consistent. The expression of *SlAN1* in young tomato plants and *PhAN1* in petunia plants was increased under high light exposure (Albert et al., 2014; Kiferle et al., 2015) while Lightbourn et al. (2007) did not observe any significant change in transcription of *CaAN1* in pepper leaves after applying additional light. The effect of light quality on anthocyanin biosynthetic genes has hardly been studied in *Solanaceous* vegetables, only in petunia flowers, in which blue and red light were reported to induce the expression of *CHS* genes when compared to dark condition (Katz and Weiss, 1999). Studies in *Arabidopsis* and other plants provided more evidence for the stimulatory effect of blue and red light on anthocyanin production by increasing the transcription of *R2R3-MYB* activator genes and structural genes (Shi et al., 2014; Xu et al., 2014; Liu Z. et al., 2015).

**Figure 8:**
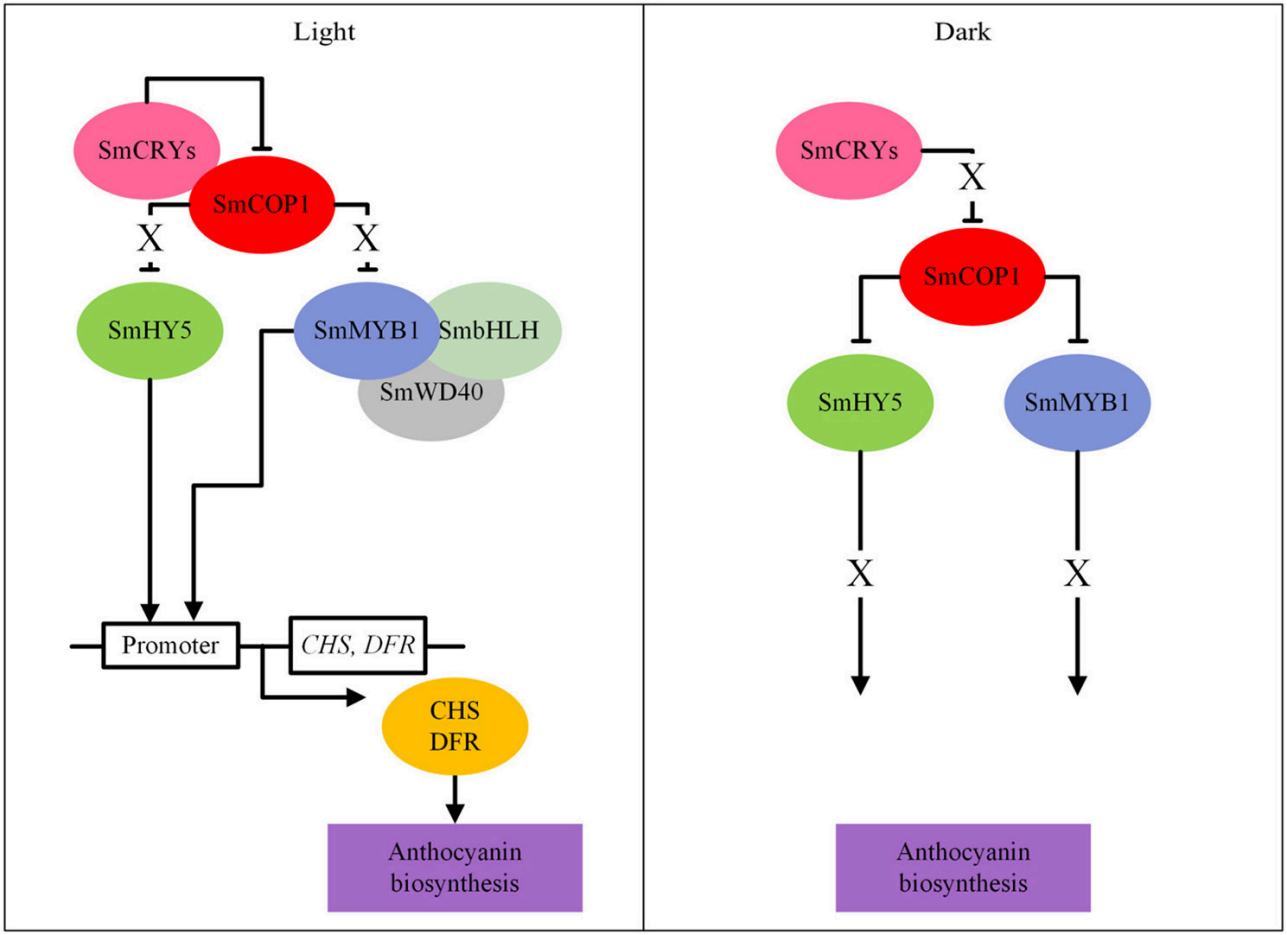
A model for light-dependent anthocyanin biosynthesis in eggplants (based on Jiang et al., 2016b). The “ → ” means activation, “—|” means repression and “X” means inactivation.

Anthocyanin biosynthetic genes were upregulated under light and downregulated under darkness in tobacco leaves transiently overexpressing the potato *StMYBA1*gene, under control of the *CaMV* 35S promoter (Liu et al., 2017). This suggests that, in addition to the right genetic makeup, light is an important cue for anthocyanin production in *Solanaceae*. Application of light-impermeable bagging to eggplant during cultivation resulted in white fruits. Jiang et al. (2016b) investigated the role of several light-signal transduction components in the light-dependent regulation of anthocyanin biosynthesis in eggplant. They studied the protein-protein interactions of SmCOP1 (a repressor of photomorphogenesis and anthocyanin biosynthesis), SmHY5 (a BZIP transcription factor promoting expression of light-inducible genes, such as anthocyanin biosynthetic genes), SmCRY1 and SmCRY2 (two blue light photoreceptors) and SmMYB1, by yeast two-hybrid and bimolecular fluorescence complementation analyses. They identified interactions between SmCRYs and SmCOP1, between SmCOP1 and SmHY5 and between SmCOP1 and SmMYB1. Based on these interactions, Jiang proposed a model for light-induced anthocyanin biosynthesis in eggplant (Figure 8): in light, SmCRYs inhibited the activity of SmCOP1, which allowed SmHY5 and SmMYB1 to bind to the promoters of *SmCHS* and *SmDFR* genes resulting in anthocyanin biosynthesis in eggplant; in darkness, SmCRYs failed to inhibit the function of SmCOP1 and consequently, SmHY5 and SmMYB1 were targeted by SmCOP1 for ubiquitination and subsequent protein degradation through a 26S proteasome pathway, thus blocking the MYB1-dependent activation of anthocyanin biosynthesis. This model nicely demonstrates that, in addition to transcriptional regulation, post-translational control mechanisms also play an important role in regulating the anthocyanin pathway.

### Temperature

Temperature is another major environmental factor influencing anthocyanin metabolism. Low temperature induced anthocyanin accumulation in *Solanaceae* (Løvdal et al., 2010; Jiang et al., 2016a). Jaakola (2013) and Xu et al. (2015) proposed that the regulation of anthocyanin biosynthesis by low temperature and light might be through the same mechanism, as induction of anthocyanin biosynthesis at low temperature needed light. Nevertheless, the mechanism is not fully understood. Several transcription factors, including SlAN2, SlAN1, and SlJAF13 mediated anthocyanin biosynthesis under low temperature (Kiferle et al., 2015; Qiu et al., 2016). Structural genes *SlCHS, SlF3H, SlF3*′*5*′*H*, and *SlDFR* were upregulated in cold conditions (Løvdal et al., 2010; Kiferle et al., 2015; Qiu et al., 2016). In eggplant, EBGs (*SmCHS, SmCHI*, and *SmF3H*) have been reported to respond earlier than LBGs (*SmF3*′*5H, SmDFR*, and *SmANS*) under low temperature (Jiang et al., 2016a). The expression of *SlAN11* was neither influenced by high light nor by low temperature, suggesting that *SlAN11* expression is independent of light and temperature stimuli (Kiferle et al., 2015).

High temperature reduced anthocyanin accumulation occurs in plants by inhibiting the expression of anthocyanin activators and related structural genes and/or enhancing that of repressors (Yamane et al., 2006; Rowan et al., 2009; Lin-Wang et al., 2011). For example, from veraison to harvest stage, both the transcriptional and enzymatic levels of anthocyanin biosynthesis were restrained in grape berries (cv. Sangiovese) at high temperature (Movahed et al., 2016). In addition, the peroxidase activity in these berries increased. Movahed et al. (2016) overexpressed *VviPrx31*, encoding a grapevine class III peroxidase, in petunia and caused anthocyanin reduction in petunia petals under heat stress, indicating active anthocyanin degradation. It further indicated that VviPrx31 is responsible for anthocyanin degradation at high temperature. Therefore, the effect of high temperature reducing anthocyanin content in grape berries is not only contributed by impairing biosynthesis, but likely also by enhancing degradation. High temperature induced anthocyanin degradation was also suggested in plum fruits (Niu et al., 2017). The high temperature-dependent decrease in anthocyanin concentration was associated with an increased activity of a class III peroxidase and elevated H_2_O_2_ levels. However, by applying peroxidase inhibitors, anthocyanin content under both temperature treatments increased and the increasing extent was even higher at 35°C compared to 20°C, despite the higher H_2_O_2_ level at high temperature. Therefore, the increased peroxidase activity was indicated to contribute to reduced anthocyanin content at high temperature. In plum fruits, the concentration of protocatechuic acid, a product resulting from H_2_O_2_ mediated oxidation of anthocyanins *in vitro*, barely changed at 20°C, but significantly increased at 35°C. This suggests that protocatechuic acid could be an anthocyanin degradation product *in vivo* due to a class III peroxidase catalyzed anthocyanin degradation by H_2_O_2_. In conclusion, anthocyanin degradation might result from the increased activity of peroxidase enzymes in response to thermal stress.

## Conclusion

Due to their attractive color, high antioxidant capacity, and positive effects on shelf-life, there is an increasing interest in uncovering the mechanism of anthocyanin metabolism in *Solanaceous* vegetables such as pepper, eggplant, tomato and potato. Numerous anthocyanin compounds, including the six most common anthocyanidin derivatives, have been found in these vegetables. Delphinidin-based anthocyanins, which have a very high antioxidant capacity, are predominantly present in purple pepper, eggplant, and tomato fruits and potato tubers, in addition to pelargonidin-based anthocyanins which are mainly present in red potato tubers. Anthocyanidin-3-(*p*-coumaroyl-rutinoside)-5-glucoside is the most abundant structure of anthocyanins in these vegetables.

Besides the qualitative variations in chemical structure, there are also quantitative variations in anthocyanin content. During fruit development, anthocyanin levels increase until they reach a maximum level prior to ripening and, in most cases, decrease when ripening progresses. Discoloration of fruits is attributed to either reduced biosynthesis or increased degradation of anthocyanins, or a combination of both. In the anthocyanin biosynthetic pathway, expression of late biosynthetic genes determines the quantitative variation in anthocyanins. Transcript levels of late biosynthetic genes decrease during later stages of ripening when discoloration occurs. Anthocyanin biosynthesis is regulated by MBW complexes consisting of different MYBs, but with the same bHLH and WD40 transcription factors. Reduced biosynthesis is controlled by downregulation of MYB activators and upregulation of MYB repressors. Positive regulation of biosynthesis has been studied in depth, while there is limited progress in investigating negative regulation in the main *Solanaceous* vegetables. Only in the model plant petunia, two MYB repressors were identified, but not in other *Solanaceae*. Degradation is likely an active process, as shown for example for color fading in flowers of petunia and *B. calycina*, from which a peroxidase that can actively degrade anthocyanins *in planta* has been suggested. No information is currently available on anthocyanin degradation in the main *Solanaceous* vegetables.

In order to increase the level of anthocyanins in *Solanaceous* vegetables, biosynthesis, stability and degradation of anthocyanins should be taken into account. Increasing the anthocyanin biosynthesis can be achieved by environmental and genetic options. Anthocyanin biosynthesis has been shown to be a light-dependent feature. As a short-term solution, environmental stimuli such as high light intensity, blue/UV light and low temperature can be applied during cultivation to promote anthocyanin production. For a long-term solution, modern breeding tools, for instance genetic engineering, can be applied to not only increase production, but also optimize anthocyanin levels through stabilizing their structure and reducing their degradation. Therefore, we need to increase our understanding of transcriptional and post-transcriptional regulation, especially how repressors function and by what mechanisms degradation occurs. Also, the links between anthocyanin degradation and environmental regulation need to be investigated further.

## Author contributions

YL did the literature research, drafted the manuscript and made tables and figures. YT, RS, LM, RV, and AB provided comments and helped in writing the final manuscript. AB improved Figure 2. YT improved Figure 5.

### Conflict of interest statement

The authors declare that the research was conducted in the absence of any commercial or financial relationships that could be construed as a potential conflict of interest. The reviewer, GT, and handling Editor declared their shared affiliation.

## References

[B1] Achterfeldt S. Traka M. Martin C. Vauzour D. Kroon P. A. (2015). Do anthocyanins in purple tomatoes reduce the risk of cardiovascular disease? Proc. Nutr. Soc. 74:E85 10.1017/S0029665115001007

[B2] Aguilar-Barragán A. Ochoa-Alejo N. (2014). Virus-induced silencing of MYB and WD40 transcription factor genes affects the accumulation of anthocyanins in chilli pepper fruit. Biol. Plant. 58, 567–574. 10.1007/s10535-014-0427-4

[B3] Ahmed N. U. Park J. I. Jung H. J. Yang T. J. Hur Y. Nou I. S. (2014). Characterization of dihydroflavonol 4-reductase (DFR) genes and their association with cold and freezing stress in *Brassica rapa*. Gene 550, 46–55. 10.1016/j.gene.2014.08.013 25108127

[B4] Albert N. W. Davies K. M. Lewis D. H. Zhang H. Montefiori M. Brendolise C.. (2014). A conserved network of transcriptional activators and repressors regulates anthocyanin pigmentation in eudicots. Plant Cell 26, 962–980. 10.1105/tpc.113.122069 24642943 PMC4001404

[B5] Albert N. W. Lewis D. H. Zhang H. Irving L. J. Jameson P. E. Davies K. M. (2009). Light-induced vegetative anthocyanin pigmentation in petunia. J. Exp. Bot. 60, 2191–2202. 10.1093/jxb/erp097 19380423 PMC2682507

[B6] Albert N. W. Lewis D. H. Zhang H. Schwinn K. E. Jameson P. E. Davies K. M. (2011). Members of an R2R3-MYB transcription factor family in petunia are developmentally and environmentally regulated to control complex floral and vegetative pigmentation patterning. Plant J. 65, 771–784. 10.1111/j.1365-313X.2010.04465.x 21235651

[B7] André C. M. Schafleitner R. Legay S. Lefèvre I. Aliaga C. A. Nomberto G.. (2009). Gene expression changes related to the production of phenolic compounds in potato tubers grown under drought stress. Phytochemistry 70, 1107–1116. 10.1016/j.phytochem.2009.07.008 19664789

[B8] Aza-Gonzalez C. Herrera-Isidron L. Nunez-Palenius H. G. De La Vega O. M. Ochoa-Alejo N. (2013). Anthocyanin accumulation and expression analysis of biosynthesis-related genes during chili pepper fruit development. Biol. Plant. 57, 49–55. 10.1007/s10535-012-0265-1

[B9] Azuma K. Ohyama A. Ippoushi K. Ichiyanagi T. Takeuchi A. Saito T.. (2008). Structures and antioxidant activity of anthocyanins in many accessions of eggplant and its related species. J. Agric. Food Chem. 56, 10154–10159. 10.1021/jf801322m 18831559

[B10] Ballester A. R. Molthoff J. de Vos R. Hekkert B. L. Orzaez D. Fernández-Moreno J. P.. (2010). Biochemical and molecular analysis of pink tomatoes: deregulated expression of the gene encoding transcription factor SLMYB12 leads to pink tomato fruit color. Plant Physiol. 152, 71–84. 10.1104/pp.109.147322 19906891 PMC2799347

[B11] Barbagallo R. N. Palmeri R. Fabiano S. Rapisarda P. Spagna G. (2007). Characteristic of β-glucosidase from Sicilian blood oranges in relation to anthocyanin degradation. Enzyme Microb. Technol. 41, 570–575. 10.1016/j.enzmictec.2007.05.006

[B12] Basílio N. Pina F. (2016). Chemistry and photochemistry of anthocyanins and related compounds: a thermodynamic and kinetic approach. Molecules 21:E1502. 10.3390/molecules21111502 27834931 PMC6273059

[B13] Bassolino L. Zhang Y. Schoonbeek H. J. Kiferle C. Perata P. Martin C. (2013). Accumulation of anthocyanins in tomato skin extends shelf life. New Phytol. 200, 650–655. 10.1111/nph.12524 24102530

[B14] Bast A. Haenen G. R. (2013). Ten misconceptions about antioxidants. Trends Pharmacol. Sci. 34, 430–436. 10.1016/j.tips.2013.05.010 23806765

[B15] Borovsky Y. Oren-Shamir M. Ovadia R. De Jong W. Paran I. (2004). The a locus that controls anthocyanin accumulation in pepper encodes a MYB transcription factor homologous to Anthocyanin2 of petunia. Theor. Appl. Gen. 109, 23–29. 10.1007/s00122-004-1625-9 14997303

[B16] Butelli E. Titta L. Giorgio M. Mock H. P. Matros A. Peterek S.. (2008). Enrichment of tomato fruit with health-promoting anthocyanins by expression of select transcription factors. Nat. Biotechnol. 26, 1301–1308. 10.1038/nbt.1506 18953354

[B17] Carocho M. Ferreira I. C. (2013). A review on antioxidants, prooxidants and related controversy: natural and synthetic compounds, screening and analysis methodologies and future perspectives. Food Chem. Toxicol. 51, 15–25. 10.1016/j.fct.2012.09.021 23017782

[B18] Chalker-Scott L. (1999). Environmental significance of anthocyanins in plant stress responses. Photochem. Photobiol. 70, 1–9. 10.1111/j.1751-1097.1999.tb01944.x

[B19] Charepalli V. Reddivari L. Radhakrishnan S. Vadde R. Agarwal R. Vanamala J. K. (2015). Anthocyanin-containing purple-fleshed potatoes suppress colon tumorigenesis via elimination of colon cancer stem cells. J. Nutr. Biochem. 26, 1641–1649. 10.1016/j.jnutbio.2015.08.005 26383537

[B20] D'Amelia V. Aversano R. Batelli G. Caruso I. Moreno M. C. Castro-Sanz A. B.. (2014). High AN1 variability and interaction with basic helix-loop-helix co-factors related to anthocyanin biosynthesis in potato leaves. Plant J. 80, 527–540. 10.1111/tpj.12653 25159050

[B21] De Jong W. S. De Jong D. M. De Jong H. Kalazich J. Bodis M. (2003). An allele of dihydroflavonol 4-reductase associated with the ability to produce red anthocyanin pigments in potato (*Solanum tuberosum* L.). Theor. Appl. Genet. 107, 1375–1383. 10.1007/s00122-003-1395-9 12955207

[B22] De Vlaming P. Vaneekeres J. E. M. Wiering H. (1982). A gene for flower color fading in Petunia-Hybrida. Theor. Appl. Genet. 61, 41–46. 10.1007/BF00261508 24271372

[B23] Dhar M. K. Sharma R. Koul A. Kaul S. (2015). Development of fruit color in Solanaceae: a story of two biosynthetic pathways. Brief. Func. Genomics 14, 199–212. 10.1093/bfgp/elu018 24916164

[B24] Docimo T. Francese G. Ruggiero A. Batelli G. De Palma M. Bassolino L.. (2016). Phenylpropanoids accumulation in eggplant fruit: characterization of biosynthetic genes and regulation by a MYB transcription factor. Front. Plant Sci. 6:1233. 10.3389/fpls.2015.01233 26858726 PMC4729908

[B25] Dubos C. Stracke R. Grotewold E. Weisshaar B. Martin C. Lepiniec L. (2010). MYB transcription factors in Arabidopsis. Trends Plant Sci. 15, 573–581. 10.1016/j.tplants.2010.06.005 20674465

[B26] Faraco M. Spelt C. Bliek M. Verweij W. Hoshino A. Espen L.. (2014). Hyperacidification of vacuoles by the combined action of two different p-atpases in the tonoplast determines flower color. Cell Rep. 6, 32–43. 10.1016/j.celrep.2013.12.009 24388746

[B27] Forkmann G. Ruhnau B. (1987). Distinct substrate specificity of dihydroflavonol 4-reductase from flowers of *Petunia hybrida*. Zeitschrift für Naturforschung C 42, 1146–1148. 10.1515/znc-1987-9-1026

[B28] Gisbert C. Dumm J. M. Prohens J. Vilanova S. Stommel J. R. (2016). A spontaneous eggplant (*Solanum melongena* L.) color mutant conditions anthocyanin-free fruit pigmentation. Hortscience 51, 793–798. Available online at: http://hortsci.ashspublications.org/content/51/7/793.short

[B29] Gonzali S. Mazzucato A. Perata P. (2009). Purple as a tomato: towards high anthocyanin tomatoes. Trends Plant Sci. 14, 237–241. 10.1016/j.tplants.2009.02.001 19359211

[B30] Gould K. S. (2003). Anthocyanins in leaves: light attenuators or antioxidants? Func. Plant Biol. 30, 865–873. 10.1071/FP03118 32689071

[B31] Gould K. S. McKelvie J. Markham K. R. (2002). Do anthocyanins function as antioxidants in leaves? Imaging of H2O2 in red and green leaves after mechanical injury. Plant Cell Environ. 25, 1261–1269. 10.1046/j.1365-3040.2002.00905.x

[B32] Guo J. Han W. Wang M. H. (2008). Ultraviolet and environmental stresses involved in the induction and regulation of anthocyanin biosynthesis: a review. Afr. J. Biotechnol. 7, 4966–4972. Available online at: https://www.ajol.info/index.php/ajb/article/view/59709

[B33] Guo J. Wang M.-H. (2010). Ultraviolet a-specific induction of anthocyanin biosynthesis and PAL expression in tomato (*Solanum lycopersicum* L.). Plant Growth Regul. 62, 1–8. 10.1007/s10725-010-9472-y

[B34] Harborne J. B. Williams C. A. (2000). Advances in flavonoid research since 1992. Phytochemistry 55, 481–504. 10.1016/S0031-9422(00)00235-1 11130659

[B35] Hernández R. Eguchi T. Deveci M. Kubota C. (2016). Tomato seedling physiological responses under different percentages of blue and red photon flux ratios using LEDs and cool white fluorescent lamps. Sci. Hortic. 213, 270–280. 10.1016/j.scienta.2016.11.005

[B36] Hoballah M. E. Gübitz T. Stuurman J. Broger L. Barone M. Mandel T.. (2007). Single gene–mediated shift in pollinator attraction in Petunia. Plant Cell 19, 779–790. 10.1105/tpc.106.048694 17337627 PMC1867374

[B37] Holton T. A. Cornish E. C. (1995). Genetics and biochemistry of anthocyanin biosynthesis. Plant Cell 7, 1071–1083. 10.1105/tpc.7.7.1071 12242398 PMC160913

[B38] Ichiyanagi T. Kashiwada Y. Shida Y. Ikeshiro Y. Kaneyuki T. Konishi T. (2005). Nasunin from eggplant consists of cis-trans isomers of delphinidin 3-[4-(p-coumaroyl)-L-rhamnosyl (1rom eggplant consists of cis-trans isomer J. Agric. Food Chem. 53, 9472–9477. 10.1021/jf051841y 16302764

[B39] Jaakola L. (2013). New insights into the regulation of anthocyanin biosynthesis in fruits. Trends Plant Sci. 18, 477–483. 10.1016/j.tplants.2013.06.003 23870661

[B40] Jia X. Shen J. Liu H. Li F. Ding N. Gao C.. (2015). Small tandem target mimic-mediated blockage of microRNA858 induces anthocyanin accumulation in tomato. Planta 242, 283–293. 10.1007/s00425-015-2305-5 25916310

[B41] Jiang M. Liu Y. Ren L. Lian H. L. Chen H. Y. (2016a). Molecular cloning and characterization of anthocyanin biosynthesis genes in eggplant (*Solanum melongena* L.). Acta Physiol. Plant. 38:163 10.1007/s11738-016-2172-0t

[B42] Jiang M. Ren L. Lian H. Liu Y. Chen H. Y. (2016b). Novel insight into the mechanism underlying light-controlled anthocyanin accumulation in eggplant (*Solanum melongena* L.). Plant Sci. 249, 46–58. 10.1016/j.plantsci.2016.04.001 27297989

[B43] Jiang Z. Chen C. Wang J. Xie W. Wang M. Li X.. (2016). Purple potato (*Solanum tuberosum* L.) anthocyanins attenuate alcohol-induced hepatic injury by enhancing antioxidant defense. J. Nat. Med. 70, 45–53. 10.1007/s11418-015-0935-3 26481011

[B44] Jiao Y. Jiang Y. Zhai W. Yang Z. (2012). Studies on antioxidant capacity of anthocyanin extract from purple sweet potato (*Ipomoea batatas* L.). Afr. J. Biotechnol. 11, 7046–7054. 10.5897/AJB11.3859

[B45] Joseph J. A. Denisova N. A. Arendash G. Gordon M. Diamond D. Shukitt-Hale B.. (2003). Blueberry supplementation enhances signaling and prevents behavioral deficits in an Alzheimer disease model. Nutr. Neurosci. 6, 153–162. 10.1080/1028415031000111282 12793519

[B46] Jung C. S. Griffiths H. M. De Jong D. M. Cheng S. Bodis M. De Jong W. S. (2005). The potato P locus codes for flavonoid 3′,5′-hydroxylase. Theor. Appl. Genet. 110, 269–275. 10.1007/s00122-004-1829-z 15565378

[B47] Jung C. S. Griffiths H. M. De Jong D. M. Cheng S. Bodis M. Kim T. S.. (2009). The potato developer (D) locus encodes an R2R3 MYB transcription factor that regulates expression of multiple anthocyanin structural genes in tuber skin. Theor. Appl. Genet. 120, 45–57. 10.1007/s00122-009-1158-3 19779693 PMC2778721

[B48] Katz A. Weiss D. (1999). Light regulation of anthocyanin accumulation and chalcone synthase gene expression in petunia flowers. Isr. J. Plant Sci. 47, 225–229. 10.1080/07929978.1999.10676777

[B49] Kiferle C. Fantini E. Bassolino L. Povero G. Spelt C. Buti S.. (2015). Tomato R2R3-MYB Proteins SlANT1 and SlAN2: same protein activity, different roles. PLoS ONE 10:e0136365. 10.1371/journal.pone.0136365 26308527 PMC4556288

[B50] Koes R. Verweij W. Quattrocchio F. (2005). Flavonoids: a colorful model for the regulation and evolution of biochemical pathways. Trends Plant Sci. 10, 236–242. 10.1016/j.tplants.2005.03.002 15882656

[B51] Kong J. M. Chia L. S. Goh N. K. Chia T. F. Brouillard R. (2003). Analysis and biological activities of anthocyanins. Phytochemistry 64, 923–933. 10.1016/S0031-9422(03)00438-2 14561507

[B52] Lachman J. Hamouz K. (2005). Red and purple coloured potatoes as a significant antioxidant source in human nutrition - a review. Plant Soil Environ. 51, 477–482. 10.17221/3620-PSE

[B53] Lachman J. Hamouz K. Šulc M. Orsák M. Pivec V. Hejtmánková A. (2009). Cultivar differences of total anthocyanins and anthocyanidins in red and purple-fleshed potatoes and their relation to antioxidant activity. Food Chem. 114, 836–843. 10.1016/j.foodchem.2008.10.029

[B54] Lachman J. Hamouz K. Orsak M. Pivec V. Hejtmankova K. Pazderu K. (2012). Impact of selected factors - Cultivar, storage, cooking and baking on the content of anthocyanins in coloured-flesh potatoes. Food Chem. 133, 1107–1116. 10.1016/j.foodchem.2011.07.077

[B55] Lee J. Lee H. K. Kim C. Y. Hong Y. J. Choe C. M. You T. W.. (2005). Purified high-dose anthocyanoside oligomer administration improves nocturnal vision and clinical symptoms in myopia subjects. Br. J. Nutr. 93, 895–899. 10.1079/BJN20051438 16022759

[B56] Lewis C. E. Walker J. R. L. Lancaster J. E. Sutton K. H. (1998). Determination of anthocyanins, flavonoids and phenolic acids in potatoes. I: coloured cultivars of *Solanum tuberosum* L. J. Sci. Food Agric. 77, 45–57. 10.1002/(SICI)1097-0010(199805)77:1<45::AID-JSFA1>3.0.CO;2-S

[B57] Li P. Castagnoli S. Cheng L. (2008). Red ‘Anjou’ pear has a higher photoprotective capacity than green ‘Anjou’. Physiol. Plant. 134, 486–498. 10.1111/j.1399-3054.2008.01155.x 18715235

[B58] Li P. Cheng L. (2008). The shaded side of apple fruit becomes more sensitive to photoinhibition with fruit development. Physiol. Plant. 134, 282–292. 10.1111/j.1399-3054.2008.01131.x 18494860

[B59] Li Q. Kubota C. (2009). Effects of supplemental light quality on growth and phytochemicals of baby leaf lettuce. Environ. Exp. Bot. 67, 59–64. 10.1016/j.envexpbot.2009.06.011

[B60] Lightbourn G. J. Griesbach R. J. Novotny J. A. Clevidence B. A. Rao D. D. Stommel J. R. (2008). Effects of anthocyanin and carotenoid combinations on foliage and immature fruit color of *Capsicum annuum* L. J. Hered. 99, 105–111. 10.1093/jhered/esm108 18222931

[B61] Lightbourn G. J. Stommel J. R. Griesbach R. J. (2007). Epistatic interactions influencing anthocyanin gene expression in *Capsicum annuum*. J. Am. Soc. Hortic. Sci. 132, 824–829. Available online at: http://journal.ashspublications.org/content/132/6/824.abstract

[B62] Lin-Wang K. Micheletti D. Palmer J. Volz R. Lozano L. Espley R.. (2011). High temperature reduces apple fruit colour via modulation of the anthocyanin regulatory complex. Plant Cell Environ. 34, 1176–1190. 10.1111/j.1365-3040.2011.02316.x 21410713

[B63] Liu Y. Lin-Wang K. Espley R. V. Wang L. Yang H. Yu B.. (2016). Functional diversification of the potato R2R3 MYB anthocyanin activators AN1, MYBA1, and MYB113 and their interaction with basic helix-loop-helix cofactors. J. Exp. Bot. 67, 2159–2176. 10.1093/jxb/erw014 26884602 PMC4809278

[B64] Liu Y. Lin-Wang K. Deng C. Warran B. Wang L. Yu B.. (2015). Comparative transcriptome analysis of white and purple potato to identify genes involved in anthocyanin biosynthesis. PLoS ONE 10:e0129148. 10.1371/journal.pone.0129148 26053878 PMC4459980

[B65] Liu Y. Wang L. Zhang J. Yu B. Wang J. Wang D. (2017). The MYB transcription factor StMYBA1 from potato requires light to activate anthocyanin biosynthesis in transgenic tobacco. J. Plant Biol. 60, 93–101. 10.1007/s12374-016-0199-9

[B66] Liu Z. Zhang Y. Wang J. Li P. Zhao C. Chen Y.. (2015). Phytochrome-interacting factors PIF4 and PIF5 negatively regulate anthocyanin biosynthesis under red light in Arabidopsis seedlings. Plant Sci. 238, 64–72. 10.1016/j.plantsci.2015.06.001 26259175

[B67] Løvdal T. Olsen K. M. Slimestad R. Verheul M. Lillo C. (2010). Synergetic effects of nitrogen depletion, temperature, and light on the content of phenolic compounds and gene expression in leaves of tomato. Phytochemistry 71, 605–613. 10.1016/j.phytochem.2009.12.014 20096428

[B68] Maier A. Hoecker U. (2015). COP1/SPA ubiquitin ligase complexes repress anthocyanin accumulation under low light and high light conditions. Plant Signal. Behav. 10:e970440. 10.4161/15592316.2014.970440 25482806 PMC4622049

[B69] Maligeppagol M. Chandra G. S. Navale P. M. Deepa H. Rajeev P. Asokan R. (2013). Anthocyanin enrichment of tomato (*Solanum lycopersicum* L.) fruit by metabolic engineering. Curr. Sci. 1, 72–80. Available online at: http://www.jstor.org/stable/24092679

[B70] Malone L. A. Barraclough E. I. Lin-Wang K. Stevenson D. E. Allan A. C. (2009). Effects of red-leaved transgenic tobacco expressing a MYB transcription factor on two herbivorous insects, *Spodoptera litura* and *Helicoverpa armigera*. Entomol. Exp. Appl. 133, 117–127. 10.1111/j.1570-7458.2009.00910.x

[B71] Mathews H. Clendennen S. K. Caldwell C. G. Liu X. L. Connors K. Matheis N.. (2003). Activation tagging in tomato identifies a transcriptional regulator of anthocyanin biosynthesis, modification, and transport. Plant Cell 15, 1689–1703. 10.1105/tpc.012963 12897245 PMC167162

[B72] Matsubara K. Kaneyuki T. Miyake T. Mori M. (2005). Antiangiogenic activity of nasunin, an antioxidant anthocyanin, in eggplant peels. J. Agric. Food Chem. 53, 6272–6275. 10.1021/jf050796r 16076105

[B73] Matsui K. Umemura Y. Ohme-Takagi M. (2008). AtMYBL2, a protein with a single MYB domain, acts as a negative regulator of anthocyanin biosynthesis in Arabidopsis. Plant J. 55, 954–967. 10.1111/j.1365-313X.2008.03565.x 18532977

[B74] Matsumaru K. Kamihama T. Inada K. (1971). Effect of covering materials with different transmission properties on anthocyanin content of eggplant pericarp. Environ. Control Biol. 9, 9–15. 10.2525/ecb1963.9.3-4_9

[B75] Mazzucato A. Willems D. Bernini R. Picarella M. E. Santangelo E. Ruiu F.. (2013). Novel phenotypes related to the breeding of purple-fruited tomatoes and effect of peel extracts on human cancer cell proliferation. Plant Physiol. Biochem. 72, 125–133. 10.1016/j.plaphy.2013.05.012 23769702

[B76] Meng X. Wang J. R. Wang G. D. Liang X. Q. Li X. D. Meng Q. W. (2015). An R2R3-MYB gene, LeAN2, positively regulated the thermo-tolerance in transgenic tomato. J. Plant Physiol. 175, 1–8. 10.1016/j.jplph.2014.09.018 25437348

[B77] Mennella G. Lo Scalzo R. Fibiani M. D'Alessandro A. Francese G. Toppino L.. (2012). Chemical and bioactive quality traits during fruit ripening in eggplant (S. *melongena L.)* and allied species. J. Agric. Food Chem. 60, 11821–11831. 10.1021/jf3037424 23134376

[B78] Mes P. J. Boches P. Myers J. R. Durst R. (2008). Characterization of tomatoes expressing anthocyanin in the fruit. J. Am. Soc. Hortic. Sci. 133, 262–269.

[B79] Montefiori M. Brendolise C. Dare A. P. Lin-Wang K. Davies K. M. Hellens R. P.. (2015). In the Solanaceae, a hierarchy of bHLHs confer distinct target specificity to the anthocyanin regulatory complex. J. Exp. Bot. 66, 1427–1436. 10.1093/jxb/eru494 25628328 PMC4339601

[B80] Movahed N. Pastore C. Cellini A. Allegro G. Valentini G. Zenoni S.. (2016). The grapevine VviPrx31 peroxidase as a candidate gene involved in anthocyanin degradation in ripening berries under high temperature. J. Plant Res. 129, 513–526. 10.1007/s10265-016-0786-3 26825649

[B81] Naito K. Umemura Y. Mori M. Sumida T. Okada T. Takamatsu N. (1998). Acylated pelargonidin glycosides from a red potato. Phytochemistry 47, 109–112. 10.1016/S0031-9422(97)00520-7

[B82] Niu J. Zhang G. Zhang W. Goltsev V. Sun S. Wang J.. (2017). Anthocyanin concentration depends on the counterbalance between its synthesis and degradation in plum fruit at high temperature. Sci. Rep. 7:7684. 10.1038/s41598-017-07896-0 28794463 PMC5550432

[B83] Noda Y. Kneyuki T. Igarashi K. Mori A. Packer L. (2000). Antioxidant activity of nasunin, an anthocyanin in eggplant peels. Toxicology 148, 119–123. 10.1016/S0300-483X(00)00202-X 10962130

[B84] Oren-Shamir M. (2009). Does anthocyanin degradation play a significant role in determining pigment concentration in plants? Plant Sci. 177, 310–316. 10.1016/j.plantsci.2009.06.015

[B85] Ou L. J. Zhang Z. Q. Dai X. Z. Zou X. X. (2013). Photooxidation tolerance characters of a new purple pepper. PLoS ONE 8:e63593. 10.1371/journal.pone.0063593 23704924 PMC3660583

[B86] Passeri V. Koes R. Quattrocchio F. M. (2016). New challenges for the design of high value plant products: stabilization of anthocyanins in plant vacuoles. Front. Plant Sci. 7:153. 10.3389/fpls.2016.00153 26909096 PMC4754442

[B87] Payyavula R. S. Singh R. K. Navarre D. A. (2013). Transcription factors, sucrose, and sucrose metabolic genes interact to regulate potato phenylpropanoid metabolism. J. Exp. Bot. 64, 5115–5131. 10.1093/jxb/ert303 24098049 PMC3830490

[B88] Pojer E. Mattivi F. Johnson D. Stockley C. S. (2013). The case for anthocyanin consumption to promote human health: a review. Comp. Rev. Food Sci. Food Saf. 12, 483–508. 10.1111/1541-4337.12024 33412667

[B89] Povero G. Gonzali S. Bassolino L. Mazzucato A. Perata P. (2011). Transcriptional analysis in high-anthocyanin tomatoes reveals synergistic effect of Aft and atv genes. J. Plant Physiol. 168, 270–279. 10.1016/j.jplph.2010.07.022 20888667

[B90] Qiu Z. Wang X. Gao J. Guo Y. Huang Z. Du Y. (2016). The tomato Hoffman's anthocyaninless gene encodes a bHLH transcription factor involved in anthocyanin biosynthesis that is developmentally regulated and induced by low temperatures. PLoS ONE 11:e0151067. 10.1371/journal.pone.0151067 26943362 PMC4778906

[B91] Quattrocchio F. Verweij W. Kroon A. Spelt C. Mol J. Koes R. (2006). PH4 of Petunia is an R2R3 MYB protein that activates vacuolar acidification through interactions with basic-helix-loop-helix transcription factors of the anthocyanin pathway. Plant Cell 18, 1274–1291. 10.1105/tpc.105.034041 16603655 PMC1456866

[B92] Quattrocchio F. Wing J. Van der Woude K. Souer E. de Vetten N. Mol J.. (1999). Molecular analysis of the anthocyanin2 gene of petunia and its role in the evolution of flower color. Plant Cell 11, 1433–1444. 10.1105/tpc.11.8.1433 10449578 PMC144295

[B93] Ramsay N. A. Glover B. J. (2005). MYB-bHLH-WD40 protein complex and the evolution of cellular diversity. Trends Plant Sci. 10, 63–70. 10.1016/j.tplants.2004.12.011 15708343

[B94] Roleira F. M. Tavares-Da-Silva E. J. Varela C. L. Costa S. C. Silva T. Garrido J.. (2015). Plant derived and dietary phenolic antioxidants: anticancer properties. Food Chem. 183, 235–258. 10.1016/j.foodchem.2015.03.039 25863633

[B95] Rowan D. D. Cao M. Lin-Wang K. Cooney J. M. Jensen D. J. Austin P. T.. (2009). Environmental regulation of leaf colour in red 35S: PAP1 Arabidopsis thaliana. New Phytol. 182, 102–115. 10.1111/j.1469-8137.2008.02737.x 19192188

[B96] Sadilova E. Stintzing F. C. Carle R. (2006). Anthocyanins, colour and antioxidant properties of eggplant (*Solanum melongena* L.) and violet pepper (*Capsicum annuum* L.) peel extracts. Z. Naturforsc. C 61, 527–535. 10.1515/znc-2006-7-810 16989312

[B97] Sapir M. Oren-Shamir M. Ovadia R. Reuveni M. Evenor D. Tadmor Y.. (2008). Molecular aspects of Anthocyanin fruit tomato in relation to high pigment-1. J. Hered. 99, 292–303. 10.1093/jhered/esm128 18344529

[B98] Schreiber G. Reuveni M. Evenor D. Oren-Shamir M. Ovadia R. Sapir-Mir M.. (2012). ANTHOCYANIN1 from *Solanum chilense* is more efficient in accumulating anthocyanin metabolites than its *Solanum lycopersicum* counterpart in association with the ANTHOCYANIN FRUIT phenotype of tomato. Theor. Appl. Genet. 124, 295–307. 10.1007/s00122-011-1705-6 21947299

[B99] Shi L. Cao S. Chen W. Yang Z. (2014). Blue light induced anthocyanin accumulation and expression of associated genes in Chinese bayberry fruit. Sci. Hortic. 179, 98–102. 10.1016/j.scienta.2014.09.022

[B100] Smeriglio A. Barreca D. Bellocco E. Trombetta D. (2016). Chemistry, pharmacology and health benefits of Anthocyanins. Phytother. Res. 30, 1265–1286. 10.1002/ptr.5642 27221033

[B101] Spelt C. Quattrocchio F. Mol J. N. Koes R. (2000). Anthocyanin1 of Petunia encodes a basic helix-loop-helix protein that directly activates transcription of structural anthocyanin genes. Plant Cell 12, 1619–1631. 10.1105/tpc.12.9.1619 11006336 PMC149074

[B102] Steyn W. J. Wand S. J. E. Holcroft D. M. Jacobs G. (2002). Anthocyanins in vegetative tissues: a proposed unified function in photoprotection. New Phytol. 155, 349–361. 10.1046/j.1469-8137.2002.00482.x 33873306

[B103] Stommel J. R. Dumm J. M. (2015). Coordinated regulation of biosynthetic and regulatory genes coincides with Anthocyanin Accumulation in developing eggplant fruit. J. Am. Soc. Hortic. Sci. 140, 129–135.

[B104] Stommel J. R. Lightbourn G. J. Winkel B. S. Griesbach R. J. (2009). Transcription factor families regulate the Anthocyanin biosynthetic pathway in *Capsicum annuum*. J. Am. Soc. Hortic. Sci. 134, 244–251.

[B105] Stushnoff C. Ducreux L. J. Hancock R. D. Hedley P. E. Holm D. G. McDougall G. J.. (2010). Flavonoid profiling and transcriptome analysis reveals new gene-metabolite correlations in tubers of *Solanum tuberosum* L. J. Exp. Bot. 61, 1225–1238. 10.1093/jxb/erp394 20110266 PMC2826661

[B106] Su X. Xu J. Rhodes D. Shen Y. Song W. Katz B.. (2016). Identification and quantification of anthocyanins in transgenic purple tomato. Food Chem. 202, 184–188. 10.1016/j.foodchem.2016.01.128 26920283

[B107] Tanaka Y. Brugliera F. (2013). Flower colour and cytochromes P450. Philos. Trans. R. Soc. B Biol. Sci. 368:20120432. 10.1098/rstb.2012.0432 23297355 PMC3538422

[B108] Tanaka Y. Ohmiya A. (2008). Seeing is believing: engineering anthocyanin and carotenoid biosynthetic pathways. Curr. Opin. Biotechnol. 19, 190–197. 10.1016/j.copbio.2008.02.015 18406131

[B109] Tanaka Y. Sasaki N. Ohmiya A. (2008). Biosynthesis of plant pigments: anthocyanins, betalains and carotenoids. Plant J. 54, 733–749. 10.1111/j.1365-313X.2008.03447.x 18476875

[B110] Toppino L. Barchi L. Lo Scalzo R. Palazzolo E. Francese G. Fibiani M.. (2016). Mapping quantitative trait loci affecting biochemical and morphological fruit properties in eggplant (*Solanum melongena* L.). Front. Plant Sci. 7:256. 10.3389/fpls.2016.00256 26973692 PMC4777957

[B111] Tsuda T. (2012). Dietary anthocyanin-rich plants: biochemical basis and recent progress in health benefits studies. Mol. Nutr. Food Res. 56, 159–170. 10.1002/mnfr.201100526 22102523

[B112] Vaknin H. Bar-Akiva A. Ovadia R. Nissim-Levi A. Forer I. Weiss D.. (2005). Active anthocyanin degradation in *Brunfelsia calycina* (yesterday-today- tomorrow) flowers. Planta 222, 19–26. 10.1007/s00425-005-1509-5 15918029

[B113] Verweij W. Spelt C. Di Sansebastiano G. P. Vermeer J. Reale L. Ferranti F.. (2008). An H+ P-ATPase on the tonoplast determines vacuolar pH and flower colour. Nat. Cell Biol. 10, 1456–1462. 10.1038/ncb1805 18997787

[B114] Verweij W. Spelt C. E. Bliek M. de Vries M. Wit N. Faraco M.. (2016). Functionally similar WRKY proteins regulate vacuolar acidification in Petunia and hair development in Arabidopsis. Plant Cell 28, 786–803. 10.1105/tpc.15.00608 26977085 PMC4826004

[B115] Wada T. Kunihiro A. Tominaga-Wada R. (2014). Arabidopsis CAPRICE (MYB) and GLABRA3 (bHLH) Control Tomato (*Solanum lycopersicum*) Anthocyanin Biosynthesis. PLoS ONE 9:e109093. 10.1371/journal.pone.0109093 25268379 PMC4182634

[B116] Watson J. (2013). Oxidants, antioxidants and the current incurability of metastatic cancers. Open Biol. 3:120144. 10.1098/rsob.120144 23303309 PMC3603456

[B117] Watson R. R. Schönlau F. (2015). Nutraceutical and antioxidant effects of a delphinidin-rich maqui berry extract Delphinol®: a review. Minerva Cardioangiol. 63, 1–12. 25892567

[B118] Woodward G. Kroon P. Cassidy A. Kay C. (2009). Anthocyanin stability and recovery: implications for the analysis of clinical and experimental samples. J. Agric. Food Chem. 57, 5271–5278. 10.1021/jf900602b 19435353

[B119] Xu F. Cao S. Shi L. Chen W. Su X. G. Yang Z. (2014). Blue light irradiation affects Anthocyanin content and enzyme activities involved in postharvest strawberry fruit. J. Agric. Food Chem. 62, 4778–4783. 10.1021/jf501120u 24783962

[B120] Xu W. Dubos C. Lepiniec L. (2015). Transcriptional control of flavonoid biosynthesis by MYB-bHLH-WDR complexes. Trends Plant Sci. 20, 176–185. 10.1016/j.tplants.2014.12.001 25577424

[B121] Yamane T. Seok T. J. Goto-Yamamoto N. Koshita Y. Kobayashi S. (2006). Effects of temperature on anthocyanin biosynthesis in grape berry skins. Am. J. Enol. Vitic. 57, 54–59.

[B122] Zhang Y. Butelli E. De Stefano R. Schoonbeek H. J. Magusin A. Pagliarani C.. (2013). Anthocyanins double the shelf life of tomatoes by delaying overripening and reducing susceptibility to gray mold. Curr. Biol. 23, 1094–1100. 10.1016/j.cub.2013.04.072 23707429 PMC3688073

[B123] Zhang Y. Butelli E. Martin C. (2014a). Engineering anthocyanin biosynthesis in plants. Curr. Opin. Plant Biol. 19, 81–90. 10.1016/j.pbi.2014.05.011 24907528

[B124] Zhang Y. Cheng S. De Jong D. Griffiths H. Halitschke R. De Jong W. (2009). The potato R locus codes for dihydroflavonol 4-reductase. Theor. Appl. Genet. 119, 931–937. 10.1007/s00122-009-1100-8 19588118 PMC2729421

[B125] Zhang Y. Hu Z. Chu G. Huang C. Tian S. Zhao Z.. (2014b). Anthocyanin accumulation and molecular analysis of anthocyanin biosynthesis-associated genes in eggplant (*Solanum melongena* L.). J. Agric. Food Chem. 62, 2906–2912. 10.1021/jf404574c 24654563

[B126] Zhang Z. Pang X. Ji Z. Jiang Y. (2001). Role of anthocyanin degradation in litchi pericarp browning. Food Chem. 75, 217–221. 10.1016/S0308-8146(01)00202-3

[B127] Zhang Z. Q. Pang X. Q. Duan X. W. Ji Z. L. Jiang Y. M. (2005). Role of peroxidase in anthocyanin degradation in litchi fruit pericarp. Food Chem. 90, 47–52. 10.1016/j.foodchem.2004.03.023

[B128] Zhao C. L. Chen Z. J. Bai X. S. Ding C. Long T. J. Wei F. G.. (2014). Structure-activity relationships of anthocyanidin glycosylation. Mol. Divers. 18, 687–700. 10.1007/s11030-014-9520-z 24792223

[B129] Zhao C. L. Guo H. C. Dong Z. Y. Zhao Q. (2009). Pharmacological and nutritional activities of potato anthocyanins. Afr. J. Pharm.Pharmacol. 3, 463–468.

[B130] Zhu H. F. Fitzsimmons K. Khandelwal A. Kranz R. G. (2009). CPC, a single-repeat R3 MYB, is a negative regulator of anthocyanin biosynthesis in arabidopsis. Mol. Plant 2, 790–802. 10.1093/mp/ssp030 19825656

[B131] Zhu H. Zhang T.-J. Zheng J. Huang X.-D. Yu Z.-C. Peng C.-L. (2017). Anthocyanins function as a light attenuator to compensate for insufficient photoprotection mediated by nonphotochemical quenching in young leaves of Acmena acuminatissima in winter. Photosynthetica, 1–10. 10.1007/s11099-017-0740-1

[B132] Zipor G. Duarte P. Carqueijeiro I. Shahar L. Ovadia R. Teper-Bamnolker P.. (2015). In planta anthocyanin degradation by a vacuolar class III peroxidase in *Brunfelsia calycina* flowers. New Phytol. 205, 653–665. 10.1111/nph.13038 25256351

